# Harnessing bacterial immunity: CRISPR-Cas system as a versatile tool in combating pathogens and revolutionizing medicine

**DOI:** 10.3389/fcimb.2025.1588446

**Published:** 2025-05-30

**Authors:** Mushtak T. S. Al-Ouqaili, Amna Ahmad, Noor A. Jwair, Farah Al-Marzooq

**Affiliations:** ^1^ Department of Microbiology, College of Medicine, University of Anbar, Ramadi, Anbar Governorate, Iraq; ^2^ Department of Microbiology and Immunology, College of Medicine and Health Sciences, United Arab Emirates University, Al-Ain, United Arab Emirates; ^3^ Anbar Health Directorate, Department of Public Health, Anbar Governorate, Ramadi, Iraq

**Keywords:** gene editing, CRISPR-Cas, Cas9, innovative therapeutics, infections

## Abstract

Clustered Regularly Interspaced Short Palindromic Repeats (CRISPR) technology has emerged as an adaptable instrument for several uses. The CRISPR–Cas system employs Cas proteins and programmable RNA molecules to guide the recognition and cleavage of specific DNA regions, permitting accurate genome editing. It is derived from the bacterial immune system and allows for accurate and efficient modification of DNA sequences. This technique provides unparalleled gene editing, control, and precise alteration opportunities. This review aims to offer a comprehensive update of the core concepts of the CRISPR–Cas system and recent progress, while also providing an overview of the significant applications in diverse fields such as microbiology and medicine. The CRISPR–Cas9 gene editing technique has facilitated substantial advancements in comprehending gene function, simulating diseases, and creating innovative therapeutics. CRISPR-based therapeutics present a hopeful prospect for addressing intricate ailments, including genetic disorders, malignancies, and infectious diseases, as they serve as viable substitutes for conventional pharmaceuticals. In microbiology, this method serves as a diagnostic and therapeutic tool that proves highly efficient in eliminating bacteria that have developed resistance to various antibiotics. Despite its significant potential, CRISPR encounters ethical, safety, and regulatory obstacles that necessitate meticulous deliberation. Concerns regarding off-target effects, poor delivery to target tissues, and unwanted side effects emphasize the necessity to thoroughly examine the technology. It is necessary to balance the advantages and difficulties CRISPR presents. Consequently, more rigorous preclinical and clinical experiments are essential before using it in humans.

## 1 Introduction to CRISPR–Cas systems

In 1987, Yoshizumi Ishino and his team from Osaka University in Japan were the first to identify CRISPR in *E. coli*, announcing the existence of CRISPR in bacteria. Subsequently, Francisco Mojica, a Spanish scientist, together with his colleagues, characterized the CRISPR sequence and introduced the term “clustered regularly interspaced short palindromic repeats” (CRISPR) to refer to it as a bacterial immune system (Jacinto et al., 2020). However, the functionality and importance of CRISPR in prokaryotes were realized later in the mid-2000s, leading to the development of a revolutionary genetic engineering tool. Emmanuelle Charpentier and Jennifer Doudna discovered CRISPR–Cas by chance while studying how bacteria defend against viruses. This tool, often described as a pair of genetic scissors, allows for the precise editing of genes. The inventors realized that this natural process could be harnessed to cut out faulty genes and replace them with healthy genes, paving the way for the future of medicine (Strzyz, 2020). The inventors documented the existence of a second short RNA, known as the trans activator CRISPR RNA (tracrRNA), which plays a vital role in the CRISPR–Cas system. They effectively reconstructed all the essential CRISPR–Cas chemicals for precise cutting of the target and suggested merging CRISPR RNA (crRNA) and tracrRNA into a single guide RNA (sgRNA). This was the first suggestion that these tools could be used for RNA-programmable genome editing through RNA-guided DNA cutter cleavage systems (Kim and Kim, 2014). Doudna also contributed to understanding protein structures involved in the DNase activity and RNA processing of the CRISPR–Cas system. In recognition of their ground-breaking work, the 2020 Nobel Prize in Chemistry was awarded to them (Uyhazi and Bennett, 2021). Together, they became the first women to share a Nobel Prize for their work in discovering and transforming CRISPR into a gene-editing technology (Ledford and Callaway, 2020). Since the discovery of this system, vast advances in technology have enabled its application in various fields of biotechnology and medicine, including microbiology. Therefore, this review aims to provide a snapshot of the recent advances of this innovative tool and its various applications.

## 2 Overview of CRISPR–Cas system and its components

CRISPR–Cas system enables accurate and targeted modification of genes, granting unparalleled authority in changing genetic information (Adli, 2018). CRISPR is an abbreviation for “Clustered Regularly Interspaced Short Palindromic Repeats,” which refers to a specific sequence of DNA in prokaryotes, found in 88% of archaea and 39% of bacteria, in both Gram-positive and Gram-negative bacteria (Barrangou, 2015). These sequences demonstrate palindromic repeats, meaning they read the same from 5’ to 3’ on one DNA strand and from 3’ to 5’ on the complementary strand (Tsui and Li, 2015). Bacteria employ a defense mechanism using CRISPR to recognize foreign DNA elements, which are similar to adaptive immunity in humans. The unique sequences between the palindromic repeats, called spacers, which are fragments taken from foreign DNA and stored in the CRISPR system. These spacers are derived from mobile genetic elements (MGEs), such as bacteriophages, transposons, or plasmids, that have previously infected bacteria (Hille and Charpentier, 2016). The identification of the spacers in the CRISPR system by sequencing provided evidence supporting the concept that bacteria may use this mechanism as a defense to detect foreign DNA fragments (Bikard and Barrangou, 2017).

**Figure 1:**
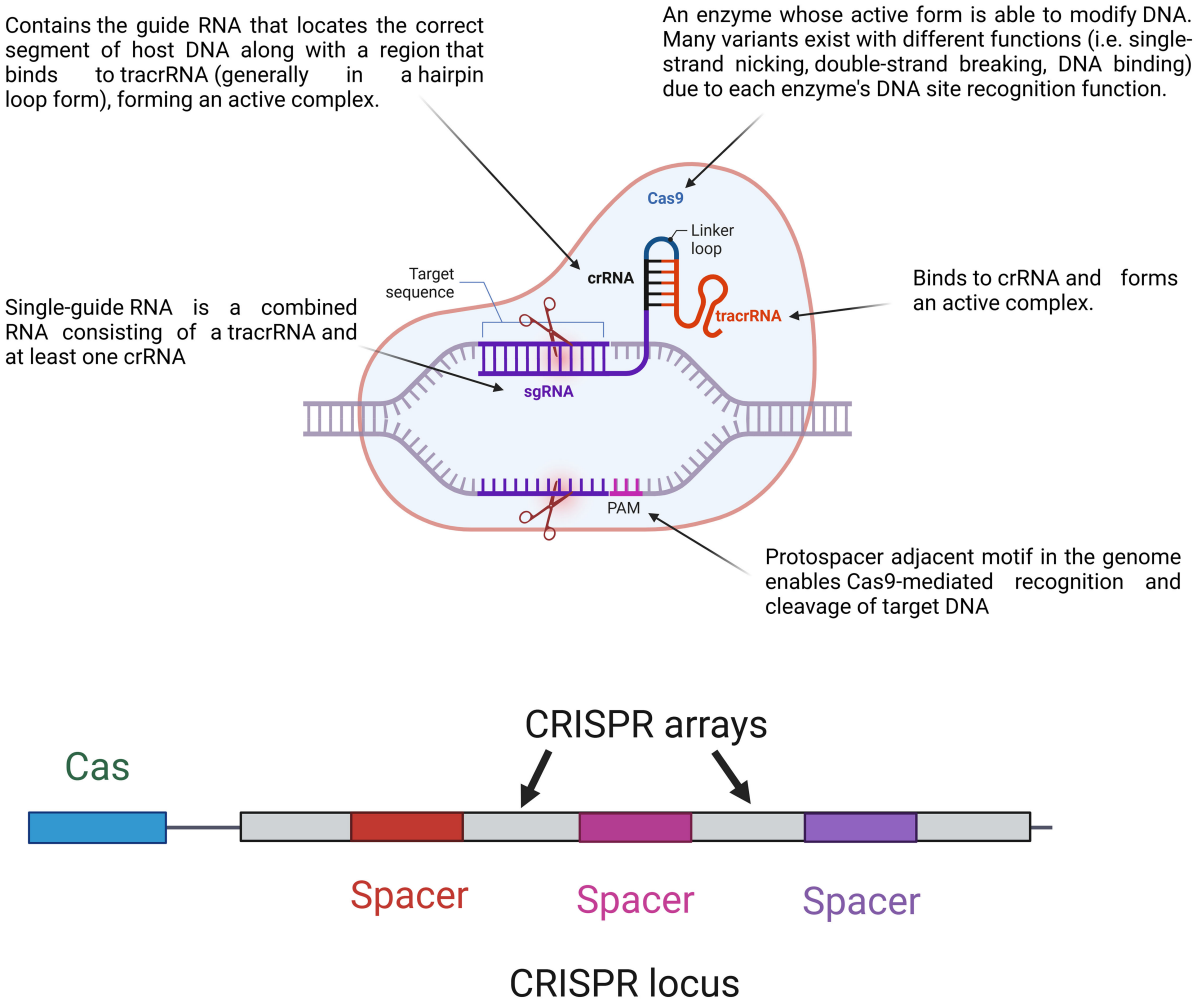
Major components of the CRISPR–Cas system and their loci. The CRISPR locus contains *cas* genes and CRISPR arrays made up of repeat and spacer sequences. During immunity, a single guided RNA (sgRNA), which combines a crRNA and tracrRNA, guides the Cas9 protein to the target DNA sequences. Cas9 forms an active complex with the sgRNA and recognizes the protospacer adjacent motif (PAM) on the target DNA, enabling precise DNA cleavage. This mechanism allows for targeted genome editing by directing Cas9 to specific DNA sites for modification.

Cas, an abbreviation for “CRISPR-associated protein,” is an enzyme that uses CRISPR sequences to recognize and impair particular DNA strands that are complementary to the ones present in the CRISPR spacer sequences (as shown in **Figure 1**) (Zhang et al., 2014). The complex formed by the Cas protein and CRISPR sequences is known as CRISPR–Cas, which can be used to defend against foreign genomes. The CRISPR–Cas adaptive immune system is used naturally by bacteria to protect themselves from foreign DNA originating from bacteriophage invasion, conjugation, or transformation (Azangou-Khyavy et al., 2020). Thus, these sequences play a significant role as weapons in fighting against foreign genes to support adaptive bacterial immunity (Barrangou et al., 2007).

The CRISPR–Cas system components are shown in **Figure 1**.

As shown in **Figure 1**, the CRISPR locus (28–37 bp) contains repeats of palindromic DNA, with a leader rich in AT (Hille and Charpentier, 2016). The spacer (32–38 bp) is located between DNA repeats and contains a piece of foreign genome (e.g., viral genome) that previously invaded the bacteria (Barrangou and Marraffini, 2014). Cas genes are located near the CRISPR locus, giving Cas enzymes their position. There are fewer than 50 units of CRISPR arrays and spacers in bacteria (Tyson and Banfield, 2008). Apart from CRISPR arrays, Cas genes have short sequences. There are ninety-three Cas genes in all, grouped into thirty-five families based on how similar the enzymes they encode are to one another. Eleven of the 35 families are part of the Cas core, which is made up of the enzymes Cas1 through Cas9 (Koonin and Makarova, 2019). CRISPR–Cas systems are divided into two categories. Class 1 systems use a combination of numerous Cas proteins to destroy the invading genome, whereas class 2 systems use a single large Cas protein for this purpose. Types II, V, and VI are further categorized into class 2, while types I, III, and IV are categorized into class 1 (Wright et al., 2016). The six groups are further divided into 19 subtypes (Westra et al., 2016). The CRISPR–Cas systems are comprised of three unique types, namely types I, II, and III. The classification is determined by the unique genes that each type possesses. Cas3, Cas9, and Cas10 are correspondingly present in types I, II, and III. Recognizing that all types and subtypes of CRISPR systems consistently contain the proteins Cas1 and Cas2 is crucial. These two proteins are essential for preserving the function of spacers (Shabbir et al., 2016). The most important types of CRISPR systems are listed in **Table 1**.

**Table 1:** Major and minor CRISPR–Cas variants (types and subtypes), their presumed roles, and signature genes. Known functions are listed for each type and subtype, while some are not well defined (ND).

Class	Cas type	Cas subtype	Signature protein (s)	Function
1	I	–	Cas3	The HD domain of single-stranded DNA nuclease and ATP-dependent helicase
		I-A	Cas8a, Cas5	Cas8, PAM recognition and targeting foreign DNA Cas5 crRNA processing/maintenance
		I-B	Cas8b	PAM recognition/targeting
		I-C	Cas8c	
		I-D	Cas10d	Contains region similar to palm domain of nucleic acid polymerases/nucleotide cyclases
		I-E	Cse1, Cse2	PAM recognition/targeting via Cse1
		I-F	Csy1, Csy2, Csy3	PAM recognition/targeting via Csy1
		I-G	GSU0054	PAM recognition/targeting
	III	–	Cas10	Cas10d/Cse1 homolog; stabilizes interference complex by binding target RNA
		III-A	Csm2	ND
		III-B	Cmr5	
		III-C	Cas10 or Csx11	
		III-D	Csx10	
		III-E	–	
		III-F		
	IV	–	Csf1	
		IV-A	–	
		IV-B		
		IV-C		
2	II	–	Cas9	HNH & RuvC nucleases for DSBs/SSBs; facilitates spacer acquisition during adaptation
		II-A	Csn2	Ring-form DNA binding protein; involved in primed adaptation
		II-B	Cas4	Endonuclease working with Cas1/Cas2 to create spacer sequences
		II-C	–	Distinguished by the lack of Csn2 or Cas4
	V	–	Cas12	RuvC nuclease activity, lacks HNH domain
		V-A	Cas12a (Cpf1)	
		V-B	Cas12b (C2c1)	
		V-C	Cas12c (C2c3)	
		V-D	Cas12d (CasY)	
		V-E	Cas12e (CasX)	
		V-F	Cas12f (Cas14, C2c10)	
		V-G	Cas12g	
		V-H	Cas12h	
		V-I	Cas12i	
		V-K	Cas12k (C2c5)	
		V-U	C2c4, C2c8, C2c9	
	VI	–	Cas13	RNA-guided RNase
		VI-A	Cas13a (C2c2)	
		VI-B	Cas13b	
		VI-C	Cas13c	
		VI-D	Cas13d	

## 3 Mechanisms and functional diversity of CRISPR–Cas system

### 3.1 Core components and functional motifs

Genetic analysis of foreign nucleic acid regions, specifically protospacers, has revealed that their elimination is not random. Usually, it is located near protospacer adjacent motifs (PAMs), which are brief DNA sequences (usually 2–6 base pairs in length) positioned one nucleotide downstream of the complementary region of the guide RNA (Mojica et al., 2009). The CRISPR system, namely CRISPR–Cas9, cleaves foreign DNA sections that are situated downstream of the PAM sequence (Vorontsova et al., 2015). Research has demonstrated that PAMs are essential for the acquisition of spacers in type I and type II CRISPR–Cas systems, but they are not required in type III systems (Sonmez, 2021). A mechanism in the CRISPR system regulates the size of the spacer when protospacers are cut near a PAM sequence (Shah et al., 2013). The PAM sequence is present in the foreign nucleic acid of viruses and plasmids, but it is lacking from the bacterial CRISPR locus since it is not a part of bacterial DNA. If the PAM sequence comes before Cas9, it cannot attach to or break the target genome. Consequently, the PAM region is required to prevent Cas enzymes from damaging the CRISPR locus (Swarts et al., 2012). Guide RNA (gRNA), a small RNA sequence not translated into protein, is required for this process. gRNA binds to a complementary foreign DNA sequence. The gRNA is composed of CRISPR RNA (crRNA) and trans-activating CRISPR RNA (tracrRNA) (Kouranova et al., 2016). Its length ranges from 17 to 24 nucleotides, and it has a GC percentage of 40-80%. Increasing this percentage strengthens the binding between the RNA and the foreign DNA (Konstantakos et al., 2022). The mechanism becomes less selective, and the RNA binds to multiple regions in the genome when the length decreases to less than 17 nucleotides (Konstantakos et al., 2022). The gRNA forms a complex with the Cas9 protein and guides it to cleave a specific location in foreign DNA using complementary base pairing between the RNA and foreign DNA (Jiang and Doudna, 2017).

The novel spacers are inserted in a specific direction in a CRISPR array (Pourcel et al., 2005), ideally situated close to the sequence of the leader. The new spacer obtained after infection is inserted between the first and second repeats of the CRISPR array in the *E. coli* type I-E system, and the first repeat next to the leader sequence is duplicated (Yosef et al., 2012).

According to Charpentier and Doudna’s 2012 study, the CRISPR gene-editing mechanism consists of a guide molecule that functions as a GPS and locates and binds to a particular gene location on a virus’s DNA. Furthermore, the DNA is cut by the CRISPR-associated protein (Cas), which functions as a molecular scissor (Westermann et al., 2021). Selective targeting of a specific DNA sequence is the first step in genome editing. Cas9 and guide RNA work together to form a complex that can recognize target sequences. Recent research has shown that Cas9 can prevent viruses from altering host DNA. Without guide RNA, Cas9 is inactive (Jinek et al., 2014). A single strand that forms a T shape with one tetraloop and two or three stem-loops makes up the guide RNA in CRISPR systems. The guide RNA’s 5’ end is made to match the target DNA sequence. Cas9 works a conformational shift that turns it from inactive to active when the guide RNA attaches to it. While the exact reason for this conformational shift is unknown, steric interactions or weakened bonds between RNA bases and protein side chains may be responsible for it (Jinek et al., 2014). Once activated, Cas9 searches for target DNA by attaching to regions that match its PAM sequence (Sternberg et al., 2014). Cas9 unwound the bases just upstream of the PAM and couples them with the correct region on the guide RNA. If the complementary region and target area precisely match, the RuvC and HNH nuclease domains of Cas9 will cut the target DNA after the third nucleotide base upstream of the PAM (Anders et al., 2014).

### 3.2 Stages of CRISPR–Cas activity

**Figure 2:**
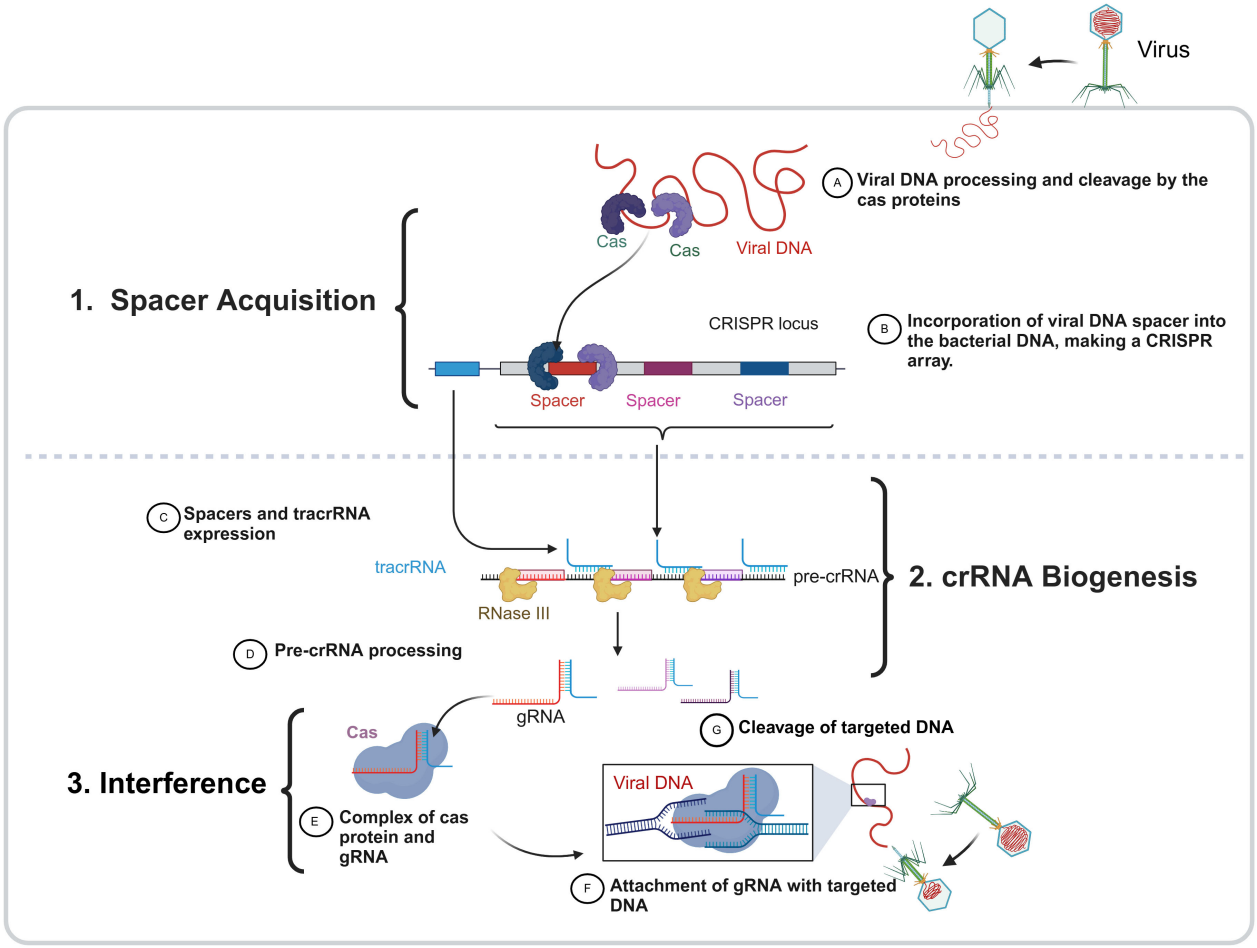
The three phases of the bacterial CRISPR–Cas function. The CRISPR-Cas immune system in bacteria operates through three main stages: (1) spacer acquisition, (2) crRNA biogenesis, (3) interference. During spacer acquisition, foreign DNA (viral DNA as an example) is recognized and cleaved by Cas proteins, and the resulting fragments are integrated into the bacterial genome at the CRISPR locus as a new spacer, forming a CRISPR array that serves as a genetic memory of past infections. In the crRNA biogenesis phase, the CRISPR array is transcribed into a precursor CRISPR RNA (pre-crRNA), which is processed by RNaseIII in the presence of a trans-activating crRNA (tracrRNA), producing mature guide RNAs (gRNAs) that carry spacer sequences. In the final interference stage, these gRNAs associate with Cas proteins to form an active surveillance complex that scans for complementary sequences in invading DNA. Upon target recognition, the Cas-gRNA complex binds to the complementary foreign DNA, leading to its cleavage and degradation.

There are three steps for CRISPR–Cas activity against foreign DNA, which are shown in **Figure 2**.

**Step 1 (spacer acquisition):** This step happens when a bacteriophage inserts nucleic acid, the initial stage involves separating a portion of the phage genome and inserting it into a CRISPR array in a spacer position between repeated palindromic sequences (sandwiched form) (Pawluk et al., 2018). This step is the same for all three types (I, II, III) when nucleic acid is first inserted by a bacteriophage into a bacterial cell. In the initial stage of the immune response, the phage genome is broken down, a small piece is removed (usually from the area near the PAM region), and this piece is inserted into a DNA spacer near the CRISPR locus. Cas genes have a role in the CRISPR process. Cas1 and Cas2 are ubiquitous in all CRISPR–Cas systems, suggesting their importance in spacer acquisition. These genes’ mutations support the theory by demonstrating that any deletion of Cas1 or Cas2 hinders the acquisition of spacers without impacting the CRISPR system (Dugar et al., 2013).

**Step 2 (crRNA processing or biogenesis):** In this step, CRISPR RNA (crRNA) is produced by the transcription of one strand of DNA that is complementary to the coding strand. Complementary sequences from both viral genome sequences and CRISPR repeats compose crRNAs (Marraffini and Sontheimer, 2010). The three distinct CRISPR system types each contain one of three forms of crRNA:

**Type I:** CRISPR repeats form loops, and the mRNA is cleaved by the Cas6e and Cas6f enzymes to produce small RNA fragments. Each fragment is composed of a CRISPR sequence as a loop and viral genome sequence, and these two fragments are referred to as crRNAs (Gesner et al., 2011).

**Figure 3:**
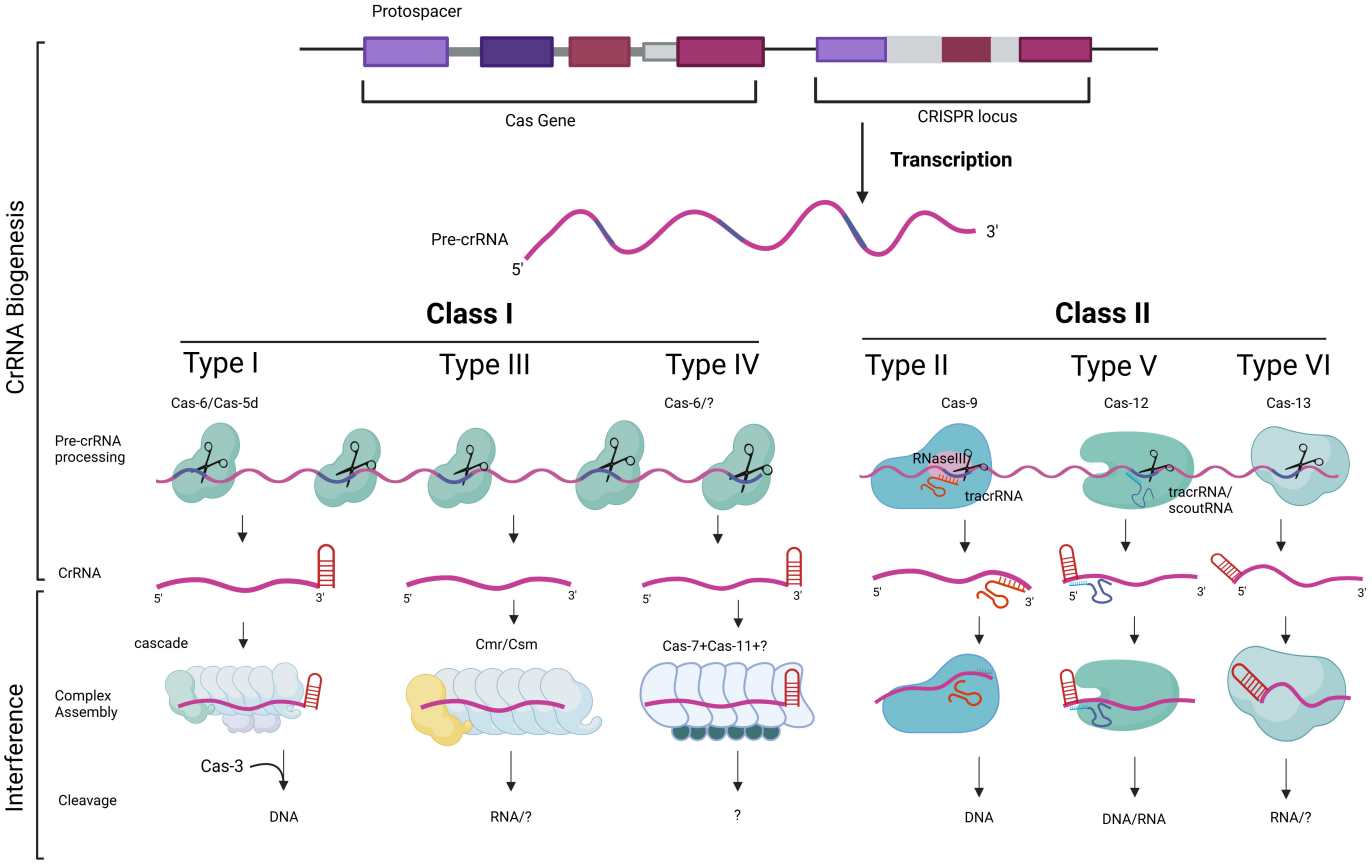
The diversity in the function of CRISPR–Cas components. The biogenesis and processing of CRISPR RNA (crRNA) and tracrRNA in CRISPR–Cas immunity are illustrated. The CRISPR-Cas system is primarily classified into Class I and Class II, based on their structural and mechanistic components. The CRISPR locus consists of repeating sequences interspaced with spacer sequences. Transcription produces a pre-crRNA which is processed by different Cas proteins depending on the CRISPR–Cas type. Class I systems (types I, III and IV) uses multi-protein complexes, such as Cas6 or Cas5d, for crRNA maturation and target recognition. Class II systems (types II, V and VI) uses a single Cas9 protein binds tracrRNA and pre-crRNA, facilitating cleavage by RNase III, resulting in mature crRNAs that guide Cas9 for targeted DNA cleavage. Types V and VI, associated with Cas12 and Cas13, respectively, use different accessory RNAs, such as tracrRNA or scoutRNA, for crRNA maturation, enabling the targeting and cleavage of DNA or RNA. This mechanistic diversity highlights the adaptability and specialization of CRISPR-Cas systems in prokaryotic immune defense.

**Type II:** In this type, there is another player called tracrRNA (shown in **Figure 3**), which is composed of multiple RNA segments bound to CRISPR repeats. The mRNA is cleaved by Cas9 and RNAseIII enzymes to produce multiple segments of CRISPR repeats and viral genome sequences along with crRNA. This complex is called tracrRNA.

**Type III**: In this type, Cas6 directly cleaves mRNA, resulting in crRNAs containing CRISPR repeats and viral genome sequences (Sashital et al., 2011).

**Step 3 (interference):** This step occurs due to the complex formation between crRNA and Cas proteins. This complex can recognize the PAM sequence in the bacteriophage genome. The presence of a PAM increases specificity because not only does the spacer recognize the PAM region, but the Cas proteins also recognize the PAM sequence (Gleditzsch et al., 2019). There is a slight difference in this step among the different types of CRISPR systems:

**Type I:** When the virus infects the bacteria for the second time, a segment of the lower strand adjacent to the PAM becomes complementary to the RNA in the CRISPR complex. This activates a cascade of Cas enzymes (**Figure 3**), which is a complex and not yet fully understood process. Ultimately, this cascade recruits Cas3 to cleave the viral genome into smaller fragments, preventing further invasion of the bacteria by the virus (Brouns et al., 2008).

**Type II:** The main player in this type is the Cas9 complex, which contains crRNA that recognizes the PAM sequence of the viral genome. Cas9 itself undergoes a double-strand break at the same site (Jinek et al., 2012).

**Type III:** This type is relatively simple, as no PAM exists. The viral genome binds to complementary RNA, and the Cas cascade degrades the viral DNA (Hale et al., 2009), as described in **Figure 3**.

When a bacteriophage (a virus that infects bacteria) attaches itself to the bacterial surface, it injects its own genome into the bacterial cell. This viral genome forces the cell to produce viral proteins and enzymes, altering the entire cell machinery. However, the CRISPR system prevents this from occurring again. Therefore, this form of memory in the CRISPR system helps avoid the same bacteriophage from invading the cell again. As such, CRISPR acts naturally as an adaptive immune system. Humans and animals have developed intricate immune systems to combat viral infections, but single-cell bacteria utilize CRISPR to identify and eliminate viral genetic material, preventing its replication (Rentmeister, 2015). The functions of CRISPR–Cas components based on their classes and types are illustrated in **Figure 3**.

## 4 CRISPR–Cas system advantages over older gene editing tools

**Figure 4:**
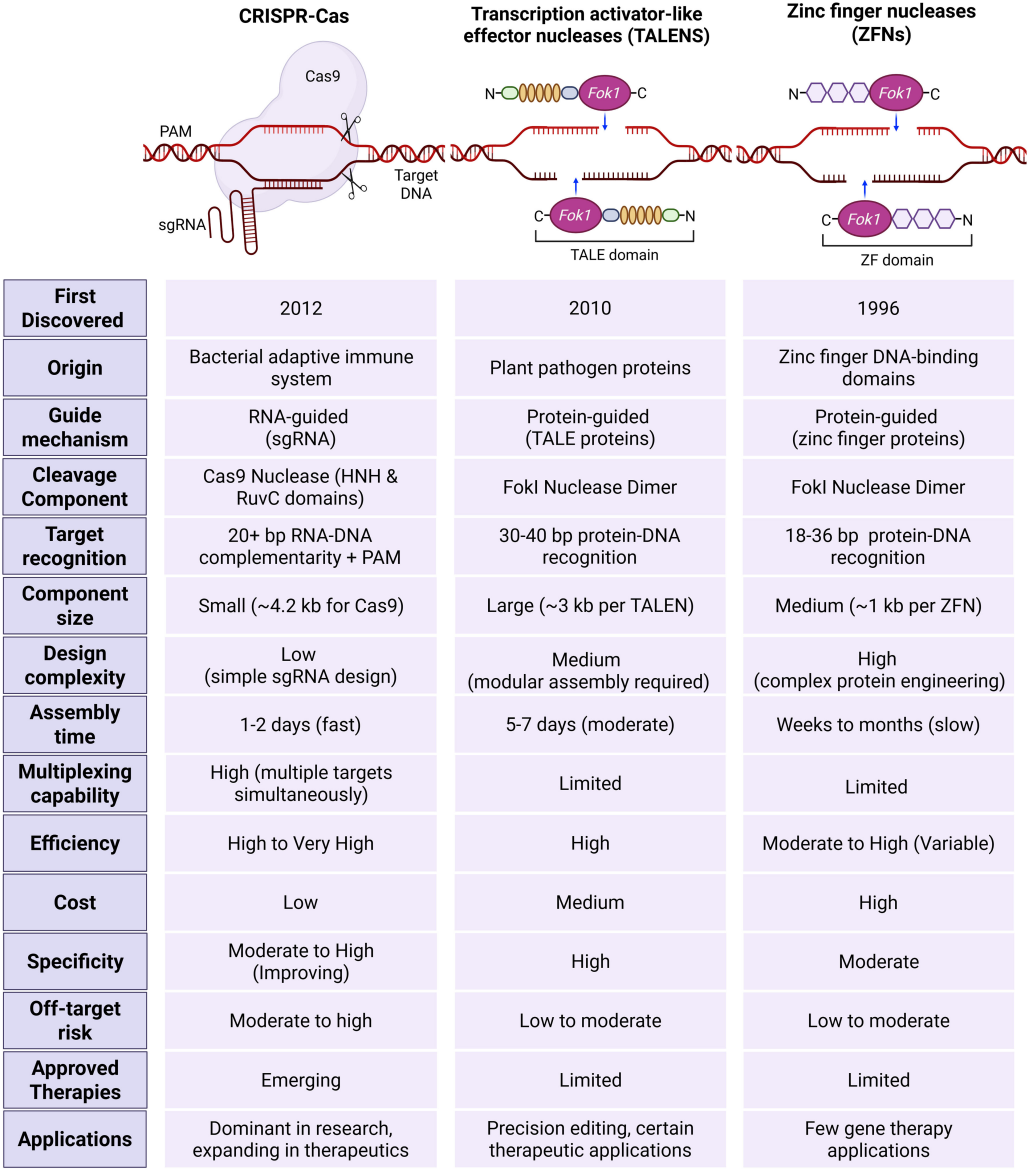
Comparison of CRISPR–Cas with other genome editing tools, including Zinc finger nucleases (ZFNs) and transcriptional activator-like effector nucleases (TALENs). CRISPR–Cas is an RNA-guided system that uses a single guide RNA to direct the Cas9 nucleases to specific DNA sequences through RNA-DNA complementarity and protospacer adjacent motif (PAM). It is characterized by a small component size, low design complexity, fast assembly time, high multiplexing capability, and high efficacy, with low cost and improved specificity. TALENs rely on protein-guided DNA recognition via TALE proteins and require the assembly of large constructs. Although TALENs offer high specificity and efficiency, they are limited in multiplexing and involve moderate design complexity and assembly time. ZFNs function through protein-DNA recognition using zinc finger domains and FokI nucleases dimers. This system demands complex protein engineering, has slower assembly, and limited flexibility. While all three systems exhibit varying degrees of off-target risk and therapeutic use, CRISPR-Cas currently dominates research and is rapidly expanding in clinical applications. In contrast, TALENs and ZFNs are primarily applied in precision editing with limited therapeutic adoption.

Programmable nucleases such as zinc finger nucleases (ZFNs) and transcriptional activator-like effector nucleases (TALENs) were used for genome editing before the CRISPR–Cas9 system’s launch. They also act as molecular scissors, which can cut DNA at the desired location within the genome, leading to targeted DNA double-strand breaks (DSBs), causing genomic modifications (Rahman et al., 2011; Bhardwaj and Nain, 2021). **Figure 4** shows the key features differentiating these three technologies. Compared with other gene editing tools, CRISPR has demonstrated superior efficiency, ease of design, cost-effectiveness, and time efficiency (Zhu, 2022).

Compared to ZFNs and TALENs, the CRISPR–Cas system has the benefit of using only single-stranded RNA (sgRNA) for editing, while other systems use proteins to identify specific genomic regions. CRISPR–Cas system stands out due to its cost-effectiveness, with potential for multiplexing. As a single Cas9 enzyme can be guided by multiple different sgRNAs supplied concurrently, researchers can target several genes or genomic sites within the same cell or organism with relative ease. This capability is extremely valuable for studying complex genetic interactions, dissecting biological pathways, engineering complex traits, or developing multi-targeted therapeutic strategies. Multiplexing with ZFNs or TALENs, requiring multiple unique protein pairs, is far more cumbersome, expensive, and less scalable (Aljabali et al., 2024). Another important feature is versatility, as the core CRISPR-Cas system is remarkably adaptable, since some Cas types, such as Cas9 protein can be modified to create variants with altered functions, leading to wider application (Villiger et al., 2024). Further, it exhibits high, robust efficiency in mediating DSBs and subsequent gene editing across a wide range of cell types and organisms. While ZFNs and TALENs can also be efficient, CRISPR often achieves high rates of modification with less optimization. Crucially, the simple and easy design, synthesis and use of sgRNA has led to the widespread acceptance of genome editing and enabled researchers to perform a wide range of genome alterations, including changing genes in living cells and species (Van Kampen and Van Rooij, 2019). These advantages lead to shortened experimental timelines from target selection to functional validation compared to the protein-based tools.

These advantages make it a powerful tool for gene editing across various fields, despite some challenges that ongoing advancements can overcome. One of these challenges is low specificity in some cases due to the short target recognition sequences as compared to the other gene editing tools, which have better specificity owing to their longer DNA recognition sites, especially for TALENs (Bhardwaj and Nain, 2021).

## 5 Applications of the CRISPR–Cas system

CRISPR–Cas systems are available in various forms and have been discovered, developed, and used to modify genes. In 2012, Jennifer Doudna and Emmanuelle Charpentier launched innovative studies by suggesting that the bacterial CRISPR–Cas9 system could serve as a customizable tool for modifying the genetic makeup of humans and other animal species. They exploited the inherent biological capacity of microorganisms and employed it in manipulating bacteria’s genetic makeup through genetic engineering (Uyhazi and Bennett, 2021). This powerful molecular scalpel allows scientists to target any desired piece of DNA and conduct genome editing. Almost any scientist can use this technology to quickly and easily alter DNA in any way they desire. Another advantage is the rapid delivery of results (Zhao et al., 2016). CRISPR’s simplicity is its distinctive beauty. It may be readily tailored to specifically target any desired gene, whether it is present in plants, animals, microorganisms, or humans.

This approach is highly significant in the fields of biotechnology and medicine due to its ability to enable accurate, cost-effective, and uncomplicated editing of genomes, both in laboratory settings and within living organisms. CRISPR has a wide range of applications, including facilitating the study of biology, aiding in diagnostics, and assisting in developing new treatments. It is also used to improve crop yields, produce biofuels, and create organs that can be transplanted (Koonin, 2018). It can be used to develop new pharmaceuticals, food products, and genetically modified organisms and to manage infections. CRISPR/Cas is also being explored for gene therapy, particularly in treating genetic diseases and cancer.

**Figure 5:**
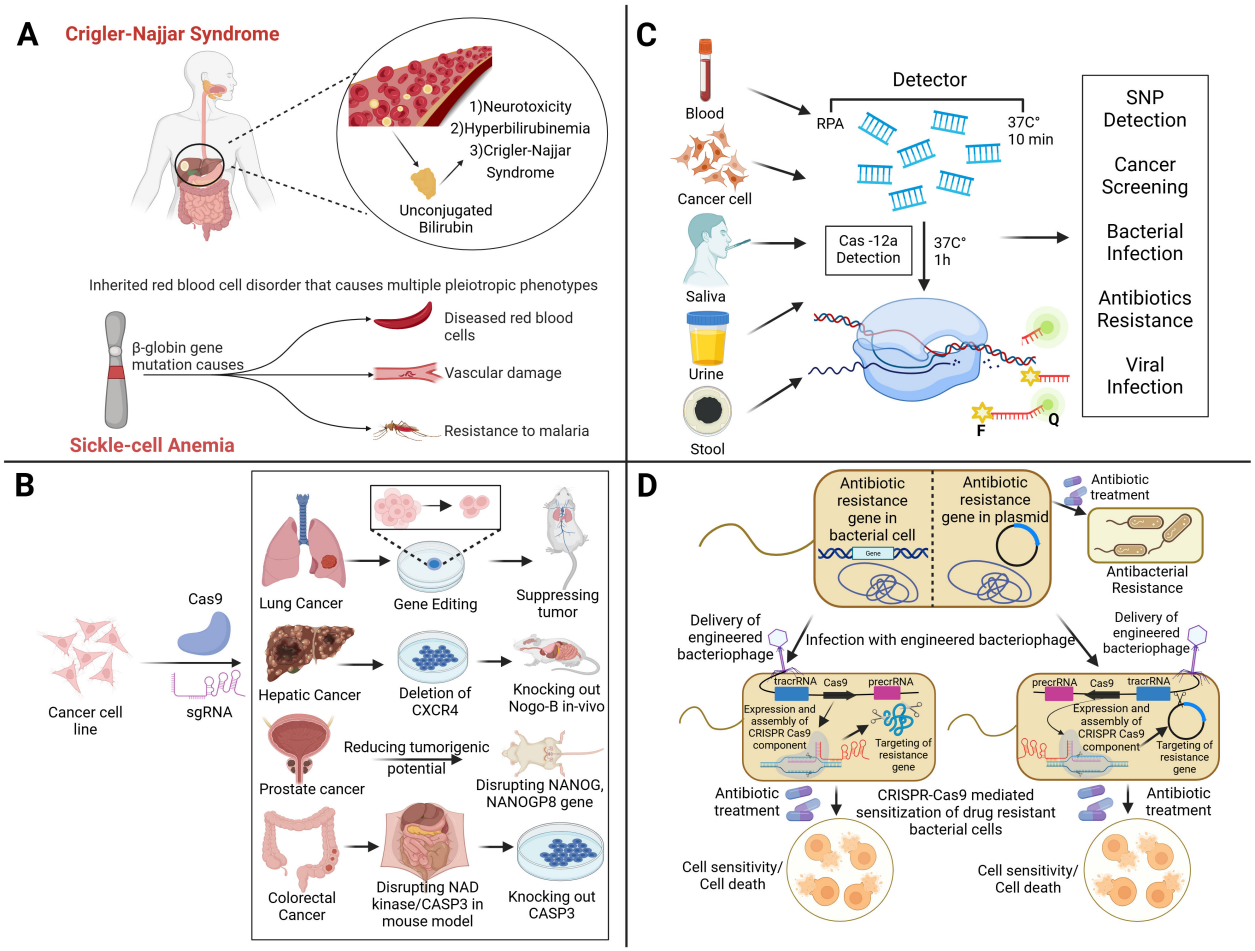
The diverse applications of CRISPR-based technologies in genetic disorders, diagnostics, cancer therapy, and antimicrobial resistance, which highlight its impact on precision medicine and infectious disease control. **(A)** CRISPR holds therapeutic potential for Crigler-Najjar Syndrome and sickle-cell anemia by targeting disease-causing mutations, such as those in β-globin gene, responsible for abnormal red blood cells and associated complications. In cancer therapy **(B)**, CRISPR-Cas9 facilitates targeted gene editing to suppress tumors by modifying oncogenic pathways, for example, by deleting CXCR4 in lung cancer, disrupting NANOG in prostate cancer, and knocking out CASP3 in colorectal cancer therapy, reducing tumorigenic potential across multiple cancer types. For diagnostics **(C)**, CRISPR-Cas12a enables rapid and sensitive detection of single-nucleotide polymorphisms (SNPs), cancer biomarkers, bacterial and viral pathogens, and antibiotic resistance markers using bodily fluids like blood, saliva, urine, and stool. In addressing antibiotic resistance **(D)**, engineered bacteriophages carrying CRISPR-Cas systems can be used to specifically target and cleave resistance genes within bacterial chromosomes or plasmids, restoring bacterial susceptibility to antibiotics and leading to cell death.

Scientists have successfully employed CRISPR technology in the laboratory to precisely alter the genes of various creatures, including fruit flies, fish, mice, plants, and even human cells (Crudele and Chamberlain, 2018). These modifications have extended beyond applications related to bacterial immune responses (Crudele and Chamberlain, 2018). Below is an up-to-date summary of various medical applications of this technology, which are also summarized in **Figure 5**.

**Table 2** provides a comprehensive overview of the most significant CRISPR-based therapies in clinical development. It summarizes the clinical trials on CRISPR-based therapies, their current stages of clinical development, and potential applications against various diseases. Since 2017, CRISPR-based therapies have achieved significant clinical milestones, highlighted by the US Food and Drug Administration (FDA) approval of the first CRISPR treatment (CASGEVY™) for sickle cell disease and β-thalassemia in 2023 (Adashi et al., 2024). *In vivo* preclinical studies have also shown potential; for instance, various CRISPR-edited CAR-T cell therapies have demonstrated promising safety and anti-tumor effects (Lei et al., 2024), with CB-010 achieving a complete response rate of 100% in early B-cell malignancy trials (Tao et al., 2024). The pipeline is steadily growing, featuring early advancements in tailored therapies such as diabetes treatments via insulin production from gene-edited islet cells (Bevacqua et al., 2024). Further preclinical developments address other conditions like Duchenne muscular dystrophy and cystic fibrosis, all focusing on somatic (non-heritable) applications rather than germline editing (Polcz and Lewis, 2016).

**Table 2:** Clinical trials on CRISPR-based therapies, target and mechanisms of each therapy, current stages of clinical development, and potential applications against various diseases.

Disease/ Condition	Therapy Name/Identifier	Target/ Mechanism	Year Started	Phase	Key Results/Status	Clinical trials	References
Sickle Cell Disease & β-thalassemia	CRISPR–Cas9-edited CD34+ cells (CTX001/exa-cel/CASGEVY)	BCL11A gene to increase fetal hemoglobin (HbF)	2018	Approved	FDA approval (Dec 2023); patients who are transfusion-independent with sustained HbF levels	NCT03745287; NCT03655678	(Frangoul et al., 2021)
Cancer (Multiple Myeloma, Solid Tumors)	CRISPR-edited T cells (CTX110, CTX120, CTX130)	Allogeneic CAR-T targeting CD19, BCMA, CD70	2019	Phase 1/2	Encouraging safety profile and anti-tumor activity	NCT04035434; NCT04244656	(Stadtmauer et al., 2020)
Cancer (Advanced Solid Tumors)	CRISPR-edited T cells	PD-1 knockout in autologous TILs	2019	Phase 1	Demonstrated feasibility and safety; modest clinical responses	NCT02793856; NCT03399448	(Stadtmauer et al., 2020)
HIV	CCR5 gene-edited CD34+ cells	CCR5 gene knockout to prevent HIV infection	2017	Phase 1/2	Demonstrated safety; limited efficacy	NCT03164135	(Xu et al., 2019)
Leber Congenital Amaurosis 10 (LCA10)	EDIT-101 (AGN-151587)	CEP290 gene correction via subretinal injection	2019	Phase 1/2	Evidence of editing, vision improvements in some patients	NCT03872479	(Maeder et al., 2019)
Transthyretin Amyloidosis	NTLA-2001	*In vivo* CRISPR to reduce TTR protein production	2020	Phase 1	Sustained reduction in serum TTR levels (>80%) after single dose	NCT04601051	(Gillmore et al., 2021)
Hereditary Angioedema	NTLA-2002	Kallikrein (KLKB1) gene knockout	2022	Phase 1/2	>90% reduction in attack rate; sustained effect	NCT05120830	(Longhurst et al., 2024)
Mucopolysaccharidosis Type I (MPS I)	SB-318	Zinc finger nuclease targeting albumin locus for IDS enzyme expression	2017	Phase 1/2	Evidence of *in vivo* genome editing; modest increases in enzyme levels	NCT02702115	(Harmatz et al., 2022; Nan et al., 2020)
Acute Myeloid Leukemia	FT819	CRISPR-engineered iPSC-derived CAR-T cells targeting CD19	2022	Phase 1	First iPSC-derived CAR-T cell therapy	NCT04629729	(Park et al., 2020)
Metastatic Gastrointestinal Cancers	Neoantigen-targeted T cells (NeoTCR-P1)	CRISPR-engineered TCR T cells targeting personalized neoantigens	2021	Phase 1	First personalized engineered TCR-T cells; demonstrated T cell persistence	NCT03970382	(Borgers et al., 2025)
Relapsed/Refractory B-cell Malignancies	CB-010	Allogeneic anti-CD19 CAR-T with additional edits for enhanced persistence	2021	Phase 1	100% CR rate in initial cohort	NCT04637763	(Nastoupil et al., 2022)
Type 1 Diabetes	VCTX210/VX-880	Gene-edited pancreatic islet cells in device	2022	Phase 1/2	Ongoing; early evidence of insulin production	NCT04786262	(Vertex, 2024)
Duchenne Muscular Dystrophy	IM-267 (formerly CRISPR-SKIP)	Exon skipping strategy targeting dystrophin	2024	Preclinical	Moving toward clinical trials	NCT not yet assigned	(Min et al., 2019)
Cystic Fibrosis	CTX003	Correction of CFTR mutations	2023	Preclinical	Moving toward clinical trials	NCT not yet assigned	(Hodges and Conlon, 2019)
Advanced Mycosis Fungoides and Sézary Syndrome	Allogeneic CD70-CAR-T cells	CRISPR-engineered CAR-T targeting CD70	2022	Phase 1	Early-stage trial	NCT04502446	(Aftimos et al., 2017)

### 5.1 CRISPR gene editing and the future of medicines

CRISPR technology is being applied across various medical domains, including the treatment of hereditary diseases, cancer, and infectious diseases. It enables personalized medicine by allowing for precise gene editing and the development of targeted therapies. Gene silencing and editing using CRISPR involves using a guide RNA that matches the DNA area of interest to lead the molecular machinery to cleave both strands of the targeted DNA. Gene silencing occurs when the cell tries to fix damaged DNA but frequently introduces mistakes that interfere with the functioning of the gene, resulting in its silence. During gene editing, a repair template that includes a precise sequence alteration is injected into the cell and incorporated into the DNA as part of the repair process. As a result, the specific DNA undergoes modifications that result in the acquisition of this new sequence (Zhang et al., 2021b).

Numerous researchers are intrigued by the prospect of employing CRISPR technology due to its encouraging preliminary results in the laboratory. There are many examples of the successful use of this approach in managing diseases, such as hereditary disorders. Several years ago, the first evidence demonstrating that CRISPR could be used to repair a faulty gene and reverse the symptoms of a disease in a living animal was published (Kannan and Ventura, 2015). For liver disease, various gene therapy approaches with specific gene targets have emerged as appealing treatment options for monogenic disorders or multifactorial disorders (Cozmescu et al., 2021). In the case of maladaptive protein expression, gene function can be disabled using the CRISPR–Cas system. For instance, blocking transthyretin can be used as a therapy for amyloidosis. Additionally, gene defects can be corrected by restoring the normal functions of liver enzymes such as fumarylacetoacetate hydrolase or alpha-1 antitrypsin (Adlat et al., 2023). Researchers have shown that a patient with a rare liver condition (Crigler–Najjar syndrome) could be cured with a single gene therapy in trials conducted on human patients (D’antiga et al., 2023). Crigler-Najjar syndrome is an inherited disorder caused by a lack of the gene UGT1A1 (as shown in **Figure 5A**). This leads to a deficiency or absence of the enzyme UDP-glucuronosyltransferase, which is necessary for the liver to convert unconjugated bilirubin into a form that can be eliminated from the body. In the subsequent study, individuals diagnosed with Crigler–Najjar syndrome successfully restored the expression of the liver enzyme UGT1A1 with the application of gene therapy.

A liver-targeting CRISPR–Cas9 delivery nanosystem was developed in a newly published study to delete the proprotein convertase subtilisin/kexin type 9 (Pcsk9) gene, which is linked to the pathophysiology of dyslipidemia. When this technique was applied in a mouse model, atherosclerosis was prevented, and cholesterol was significantly reduced (Xu et al., 2024a).

However, in the treatment of Sickle Cell disorder (SCD), CRISPR technology has emerged as a transformative tool for gene editing by reactivating fetal hemoglobin (HbF) through targeted disruption of repressor binding sites. Frati, Giacomo et al. findings showed that SCD hematopoietic stem and progenitor cells (HSPCs) exhibit higher gene editing efficiency than healthy donor cells, potentially due to differences in DNA repair mechanisms or chromatin accessibility influenced by chronic inflammation. Despite this advantage, SCD HPSCs show reduced engraftment and heightened sensitivity to cellular stress induced by the CRISPR–Cas9 procedure, underscoring the need for optimized protocols and comprehensive safety assessments. Off-targets, large deletions, and chromosomal rearrangements present challenges, highlighting the importance of high-fidelity Cas-9 variants and DSB free technologies such as base and prime editing to minimize unintended mutations. While DSB-free approaches show precision in reducing genomic stability, their efficiency in SCD cells remains limited. Moving forward, the integration of novel delivery methods, single-cell analyses, and improved editing strategies will enhance the safety and efficacy of CRISPR therapies, accelerating their transition into clinical practice to provide durable and personalized treatment for patients with SCD and other genetic disorders (Frati et al., 2024).

### 5.2 CRISPR in oncology and resistance to anti-cancer drugs

CRISPR–Cas9 has been applied experimentally to edit genomes to explore tumor occurrence, development, and metastasis, and to repair mutations or knock out specific genes involved in cancer. It has shown promise in enhancing the efficacy of cancer immunotherapy. Moreover, the application of the CRISPR–Cas9 system enables the deletion of functional domains within drug resistance genes, offering a strategy to combat anti-cancer drug resistance (**Figure 5B**). Several scientists have developed instruments that can successfully eliminate genes connected to resistance to drugs in cancer. Tyrosine kinase inhibitors (TKIs) block several targets and signaling pathways to prevent the growth and metastasis of tumors. While TKIs can be used as a treatment strategy for patients, long-term usage of them may cause resistance (Metibemu et al., 2019). Drug responsiveness may be enhanced by targeting genes linked to TKI resistance. Imatinib is one specific TKI that kills cells in conditions like gastrointestinal stromal tumors (GISTs) and chronic myeloid leukemia (CML). However, imatinib-treated cancer cells may become resistant to the medication, preventing it from killing the cells. Utilizing CRISPR gene editing technology to prevent the emergence of imatinib resistance has been covered in several studies. To increase the susceptibility of resistant cell lines linked to chronic myeloid leukemia (K562 and KCL22) to imatinib, CRISPR was utilized to repress genes including hTERT, miR-21, miRNA182, bcr-abl, and KDM6 (Zhang et al., 2021a; Shirani-Bidabadi et al., 2023). The half-maximal inhibitory concentration (IC50) of imatinib was considerably lower in K562/G01 cells when the expression of miR-21 decreased than in wild-type (WT) cells (Zhang et al., 2020). In another study, removing KDM6A increased the drug’s cellular sensitivity and reduced imatinib’s IC50 (from 1.15 to 0.24 μM) (Zhang et al., 2021a). Three K562 cell lines with decreased hTERT had GI50s (50% of maximal inhibition of cell growth) compared to the control, reducing imatinib’s efficacy (Grandjenette et al., 2020). CRISPR gene editing techniques can be used to combat resistance to additional TKI medications, including erlotinib, sorafenib, and ibritumomab. A concrete example of this can be observed in the capacity to modify the resistance to erlotinib in the non-small cell lung cancer (NSCLC) HCC827 cell line through the decrease of miR-214 expression and its subsequent target LHX6. The sensitivity to erlotinib was higher in miR-214 knockout cells (1.22 μM erlotinib IC50) and miR-214/LHX6 knockout cells (2.25 μM erlotinib) as compared to control cells (3.38 μM erlotinib IC50) (Liao et al., 2017). Several cancer types have documented resistance to antimitotic drugs such as vinca alkaloids or taxanes (Shirani-Bidabadi et al., 2023). Drug resistance has been associated with human epididymis protein-4 (HE4/WFDC2), a small secretory protein that is increased in ovarian cancer. When paclitaxel was administered to ovarian cancer cells with HE4 deletion, the survival rate of the KO cells decreased significantly; it reduced from 73.9% in normal cells to 65.9% in CRISPR-knockout cells (Ribeiro et al., 2016). Elevated Aurora-B expression in non-small cell lung cancer has been linked to cisplatin and paclitaxel resistance. To investigate this possibility, CRISPR was used to remove Aurora-B from the A549 paclitaxel-resistant (A549/PTX) cell line. Upon exposure to different doses of paclitaxel, A549/PTX WT cells exhibited increased resistance and proliferation. However, the proliferation and resistance of prA549/PTX mutant cells substantially decreased at high doses of paclitaxel. There has been talk of deleting Rsf-1, an overexpressed histone-binding protein linked to several cancers, including lung cancer, as a potential tactic to combat paclitaxel resistance. The loss of RSF1 reduced the mobility and propagation of H460 and H1299 cells and enhanced cell mortality, making them more susceptible to paclitaxel. The tumor volume was lower in the H460 cell xenograft mice treated with paclitaxel (13.0 ± 9.2 mm3) compared to the H460 cell xenograft mice treated with paclitaxel (49.4 ± 14.5 mm3). The creation of xenograft animal models employing H460 Rsf-1 KO cells served as an example of this. Atypical chemokine receptor 3 (ACKR3), a member of the G protein-coupled receptor (GPCR) superfamily, is widely expressed in a wide range of cancers, particularly in methotrexate-resistant prostate cancer tissue. The application of CRISPR-mediated ACKR3 deletion reduced the viability of DU145R and PC3R cells to around 60% and 70%, respectively, of the control when the cells were exposed to docetaxel. Doxorubicin is an anthracycline that has shown promise in treating many cancers, including breast cancer. Nevertheless, taking it could cause tumors to proliferate and treatment resistance to develop (Shirani-Bidabadi et al., 2023).

Another study introduces a novel methodology that can identify genes involved in multidrug resistance and CRISPR–Cas9 resistant cancer cell lines, and reveals critical gene networks through differential gene expression analysis. Gene regulatory network (GRN) construction using FSSEM highlights key genes like UHMK1, MGST3, and USP9X, directly linked to drug resistance, while non-coding RNAs like ESRG and LINC00664 exhibit regulatory roles in lung cancer resistance. Though none of the genes directly associated with CRISPR–Cas9 resistance were identified. However, the pathways involved in transcription and proliferation regulation show potential influences on CRISPR efficiency. The tissue-specific nature of resistance, demonstrated by the distinct GRNs for lung and intestinal cancer, underscores the importance of tailoring future research to specific cancer types. Expanding datasets and exploring non-coding gene functions will enhance understanding of resistance mechanisms, ultimately improving CRISPR-based therapies and advancing personalized cancer treatment.

### 5.3 Sharpening the edge in infectious disease diagnosis and treatment

CRISPR technology has emerged as a transformative tool in infectious disease detection and treatment. By leveraging the gene-editing capabilities of CRISPR–Cas systems, researchers are developing innovative diagnostic and therapeutic approaches that promise to enhance the management of infectious diseases (**Figures 5C, D**).

#### 5.3.1 CRISPR-based diagnostics for infections

**Figure 6:**
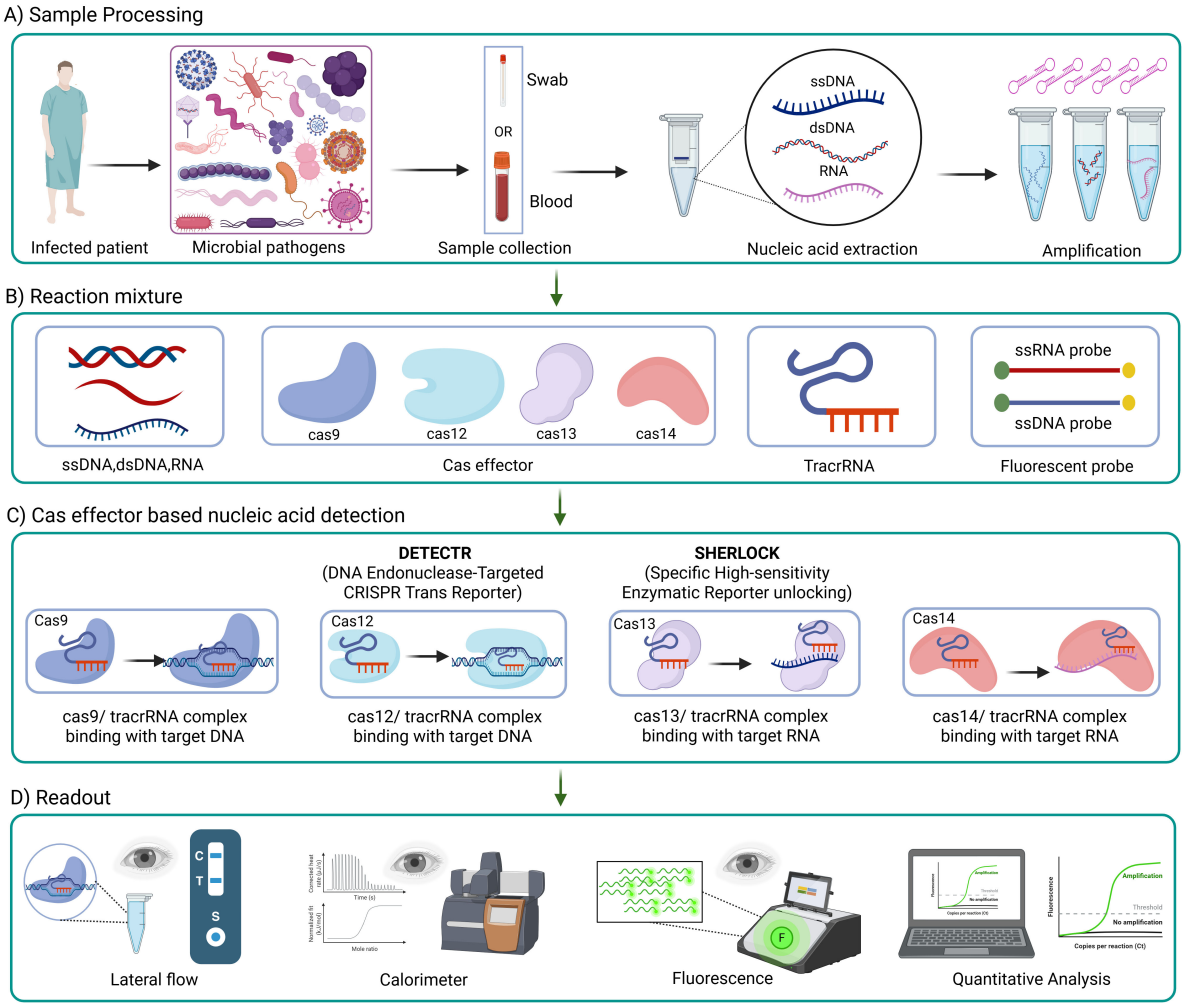
Workflow of CRISPR-based diagnostics for infections. CRISPR-based tools detects infections through four main steps; **(A)** a sample (like a swab or blood) is collected from an infected patient, and DNA or RNA is extracted and amplifies; **(B)** the reaction mixture includes Cas protein (like Cas9, Cas12, Cas13 and Cas14), guide RNAs and a reaction detector (example, fluorescent probes); **(C)** Cas proteins, paired with guide RNAs, recognize specific genetic targets, DNA or RNA, and trigger a signal using systems like DETECTR or SHERLOCK; **(D)** the results is then read using methods such as lateral flow strips, fluorescence, color changes, or computer based quantitative analysis.

CRISPR-Cas systems have been utilized to create rapid, accurate, and portable diagnostic tools that can detect infectious agents directly from clinical samples. Scientists have discovered that Cas13, a close relative of Cas9, can also be used to detect diseases. Cas13 searches for viral RNA using an RNA guide in its natural environment. Once its viral target is identified, Cas13 becomes activated (Kellner et al., 2019). In certain situations, it cuts any RNA it encounters, a process known as collateral cleavage. Researchers at the McGovern Institute, Broad Institute, and Harvard University have harnessed this mechanism to create specific high-sensitivity enzymatic reporter unlocking (SHERLOCK), an exceptionally sensitive tool for detecting human infectious diseases (as described in **Figure 6C**). Currently, SHERLOCK can be adapted to detect any genetic signature, including those associated with cancer, in virtually any location. Cas9, Cas13, and Cas12 are just a few examples of natural biological systems (shown in **Figure 6**) that scientists have modified to combat genetic and infectious diseases (Mustafa and Makhawi, 2021).

CRISPR–Cas12 system has been used to detect SARS-CoV-2, which caused the famous COVID-19 global pandemic. Scientists have developed an assay called SARS-CoV-2 DNA Endonuclease-Targeted CRISPR Trans Reporter (DETECTR). Initially, viral genomic material extracted from nasopharyngeal or oropharyngeal swabs undergoes a series of reverse transcription (as in **Figure 6A**) and isothermal amplification using loop-mediated amplification. Finally, Cas12 detects coronavirus sequences with the aid of a FAM-biotin reporter molecule, which is visualized by lateral flow strips designed to capture labeled nucleic acids (Broughton et al., 2020).

Interestingly, Cas12a-based sensing technology was also used to detect mutations in SARS-CoV-2, which represent challenges in the virus detection. Cas12a-based RT-PCR combined with a CRISPR on-site rapid detection system (RT-CORDS) platform was utilized to detect the key mutations in SARS-CoV-2 variants, such as 69/70 deletion, N501Y, and D614G. This novel assay designed type-specific crRNAs to identify wild-type (crRNA-W) and mutant (crRNA-M) virus sequences. Indeed, CRISPR tools can be helpful in the epidemiological monitoring of the spread of novel escape viral variants, besides their valuable role in clinical diagnostics (Marqués et al., 2021; He et al., 2022).

Other novel assays, such as all-in-one dual CRISPR-Cas12a (AIOD-CRISPR), were designed for ultrasensitive and visual detection of SARS-CoV-2. In the latter assay, two CRISPR RNAs without a PAM site were developed to achieve highly sensitive, specific, and robust detection of viral nucleic acids in clinical samples (Ding et al., 2020).

Technologies such as SHERLOCK and DETECTR allow ultrasensitive detection of pathogens. These innovative CRISPR-based diagnostic tests are promising as they present the potential for future development of point-of-care diagnostics, offering an alternative to conventional PCR methods. This can help rapidly identify infectious diseases without requiring extensive laboratory infrastructure (as shown in **Figure 6**). Future CRISPR-based diagnostics can be designed to be inexpensive, user-friendly, and capable of providing results quickly. The ability to perform these tests on multiple specimen types further enhances their utility in diverse clinical settings, including resource-limited areas whereby traditional diagnostics are impractical due to their need for sophisticated equipment and trained personnel.

#### 5.3.2 CRISPR-based therapeutics for infections: recent developments in CRISPR-Cas-based antimicrobials

CRISPR technology is being explored for its potential to target and cleave pathogenic DNA or RNA sequences, offering a novel approach to treating infectious diseases. Antimicrobial agents, whether synthetic or natural, are crucial substances that can effectively eliminate or hinder the growth of infectious microbes. The discovery of antibiotics has saved millions of lives; however, the increase in antibiotic resistance is causing further infectious diseases to become incurable. Antimicrobial resistance (AMR), as defined by the World Health Organization (WHO), is a worldwide problem resulting from various contributing causes, including insufficient hygiene, ineffective management, and overuse of antibiotics. This problem leads to an increase in rates of illness and mortality, an increase in expenses, and a prolongation of infectious disease durations. Conventional antimicrobial medications have become ineffective due to AMR. Producing these traditional antibiotics requires significant time and a substantial financial investment. Research on next-generation alternatives, specifically CRISPR–Cas9, has been driven by the challenges presented by existing antibacterial drugs (Getahun et al., 2022).

Shortly after the discovery of CRISPR–Cas, scientists recognized the potential of CRISPR as an antibiotic, which has recently garnered increasing attention (Aqeel and Raza, 2017; Owaid and Al-Ouqaili, 2025). The application of genome editing to the treatment of infectious diseases has the potential to revolutionize medicine. Because the essential step needed to target and cut different DNA sequences is the selective deletion of the plasmid encoding the targeted gene, CRISPR–Cas technology is especially attractive (Zhou et al., 2019). CRISPR can serve as an antibacterial agent by using either a pathogen-centric method or a gene-centric technique as described in **Figure 6D**, contingent upon the specific gene’s position. A pathogen-focused technique involves the precise targeting of specific regions on the bacterial chromosome. This method selectively eradicates the specific harmful strain and induces the demise of bacterial cells, while preserving the survival of other beneficial bacteria (Pursey et al., 2018). On the other hand, the gene-focused strategy focuses on a plasmid that contains AMR genes. This method results in plasmid removal and bacterial antibiotic re-sensitization (Tagliaferri et al., 2020). Given that AMR genes frequently migrate and have the potential to move across various bacterial species, eliminating AMR genes regardless of the host’s genetic makeup may be a successful therapy (Bhattacharyya and Mukherjee, 2020).

Since the CRISPR–Cas system is widely found in bacteria and archaea, efforts are being made to separate, enhance, and create delivery systems for this system. Researchers aim to develop RNA-guided nucleases that can efficiently target a wider variety of bacterial strains, including infections that are resistant to several drugs. Targeting particular DNA regions associated with antibiotic resistance genes and bacterial pathogens, the Cas9 nuclease is injected into microbial populations by a variety of mechanisms, such as bacteriophages, conjugative plasmid-carrying bacteria, and polymer-derivatized CRISPR nano-complexes (Gholizadeh et al., 2020).

For AMR, CRISPR–Cas can be utilized using three overarching methodologies: (i) It can target and cut genes that are particular to certain species to treat sudden illnesses. This involves using the desired bacteria while keeping the host’s microbiome unchanged (Gomaa et al., 2014; Owaid and Al-Ouqaili, 2024); (ii) The process can be targeted to cleave genes that cause medication resistance, thereby killing bacteria that carry these genes while keeping the viable wild-type susceptible clones intact, thus removing the pathogens from patients (Bikard et al., 2014); or (iii it can be employed to suppress or alter resistance genes by inducing changes that render the resistance genes non-functional while keeping the bacteria alive. This process is referred to as re-sensitization (Wang et al., 2018).

Scientists have attempted the exploitation of CRISPR–Cas system to neutralize AMR genes. This system, an effective bacterial defense mechanism, may be customized to precisely identify and cleave DNA sequences, providing a promising approach to address antibiotic resistance as described in **Figure 6D**. This approach makes use of repeat-enclosed RNA-based spacers to guide Cas proteins to DNA at particular locations, enabling the development of an adaptable tool that can target a variety of genes. Recent studies have shown that it can be lethal to intentionally or unintentionally target specific bacterial genome sequences with the CRISPR–Cas system, causing irreversible chromosomal damage and ultimately cell death (Jwair et al., 2023).

Researchers used the type I-E CRISPR–Cas system of *E. coli* in a study to specifically target crucial sites in the genomes of various strains. The findings demonstrated that it was possible to effectively eradicate bacterial strains by focusing on various sites within the genome, including individual genes. A different strategy was to remove methicillin-resistant *Staphylococcus aureus* (MRSA) strains from a mixed bacterial community by enclosing the plasmid in phage capsids, or a Cas9 phagemid. The Cas9 phagemid demonstrated efficacy in specifically targeting tetracycline-resistant plasmids and effectively decreasing the proportion of MRSA strains within the population (Brouns et al., 2008).

In another investigation, scientists introduced the Cas9 nuclease into bacteria to target specific antibiotic resistance genes using conjugative plasmids and M13-based phagemids. This technique demonstrated how the CRISPR–Cas system can distinguish between susceptible and resistant strains by significantly reducing the number of viable cells in the resistant strains. The findings highlight the potential of CRISPR-based technologies to target and eliminate antibiotic-resistant bacteria, offering a promising prospect in the ongoing fight against infections caused by bacteria (Rasheed et al., 2000, 2013).

In a study by Kang et al., 2017, a non-viral delivery method for the CRISPR–Cas system was introduced (Kang et al., 2017). The scientists employed a cationic polymer called branched polyethyleneimine (bPEI), sgRNA that targets *mecA*, and a Cas9 nano-complex. Improved Cas9 translocation into MRSA strains is demonstrated by this method. When compared to native Cas9 combined with bPEI and native Cas9 mixed with lipofectamine, a carrier that is widely used for gene delivery in mammalian cells, the Cas9-bPEI complex showed more bacterial uptake. The MRSA strains that were treated exhibited reduced growth on oxacillin-containing agar media, indicating that this technique can inhibit the development and survival of bacteria. Using Cas9 phagemids, which are plasmids made to be enclosed in phage capsids, was another important tactic. Researchers have created a CRISPR–Cas9 phagemid to target particular ARGs in MRSA strains (Bikard et al., 2014; Kang et al., 2017). Tetracycline-resistant plasmids in MRSA were effectively eradicated by the phagemid, which resulted in a marked reduction in bacterial proliferation. Similar to this, Citorik et al. (2014) used phagemids to target crucial genes in strains of bacteria resistant to antibiotics, which significantly decreased the number of viable cells (Citorik et al., 2014). These studies highlight the potential of phagemids as effective tools in CRISPR-based antimicrobial strategies. Phagemids have disadvantages despite their benefits, which include decreased plasmid content and targeted death. One disadvantage is that, once infected, phagemids do not multiply to generate other phages. Hence, a higher dosage is needed for treatment. Furthermore, their vast population and restricted host range might prevent them from being widely used. On the other hand, the use of a single nuclease with several guide RNAs to target distinct plasmids or chromosomal sequences is made possible by programmed Cas9-mediated death. This strategy demonstrates its adaptability in altering bacterial populations by potentially reducing resistant clones and minimizing the transmission of antibiotic-resistance or virulence plasmids. Moreover, the ability of the CRISPR–Cas system to modulate complex bacterial communities has been explored. Studies have demonstrated its effectiveness in selectively removing bacteria with specific genomes, reducing the prevalence of unwanted genes such as virulence loci or antibiotic resistance genes. The efficiency of Cas9 phagemids was also tested in an *in vivo* mouse model, where topical treatment significantly reduced the proportion of infected cells (Gholizadeh et al., 2020).

There have been many successful attempts to use CRISPR–Cas in managing AMR bacteria. Recent studies have reported effective eradication of AMR bacteria by specifically targeting efflux pumps (Chikkareddy, 2024). Nine specific sgRNAs were designed to target the components of the AcrAB-TolC efflux pump in *Escherichia coli* in a recent study, which showed increased susceptibility to multiple drugs, such as rifampicin, erythromycin and tetracycline, in bacterial cells with repressed efflux pump genes (Wan et al., 2022).

Moreover, several resistance genes, such as those for ciprofloxacin (*grlA, grlB*), gentamicin (*aacA*), and methicillin (*mecA*), were knocked out in MRSA using CRISPR–Cas9 technology. This study showed a noteworthy shift in the direction of antibiotic susceptibility. Another work demonstrated that pathogenic *Escherichia coli* may successfully cure *IncF* plasmids using the CRISPR–Cas9 technology. Since *IncF* plasmids contain a variety of AMR determinants, removing them from bacteria aids in the restoration of their antibiotic-susceptible status (Chen et al., 2024).

A recent study by Wang et al. introduced an innovative strategy termed as ATTACK (AssociaTes TA and CRISPR-Cas to kill) for combating multi-drug resistant (MDR) bacterial pathogens. This approach protects the CreTA module, a CRISPR regulated toxin-antitoxin (TA) system that naturally safeguards CRISPR-Cas components within the host. The underlying mechanisms are based on programmed cell death upon CRISPR-Cas inactivation. The researchers demonstrated two key mechanisms: first, the CreT component specifically targets multiple small RNAs essential for the initiation of protein synthesis; second, the CreA molecule acts as a guide RNA, directing the CRISPR-Cas complex to target CreT. The ATTACK system utilizes these elements such that, in the event of CRISPR-Cas system inactivation within MDR pathogens, CreTA is activated to induce bacterial cell death (Wang et al., 2023). Chen et al. developed an optimized CRISPR interference (CRISPRi) system based on the type I-F subtype, referred to as CSYi, for gene silencing applications in clinical isolates of *Pseudomonas Aeruginosa*. This system enabled functional characterization of resistance determinants, and notably, the researcher identified the regulatory role of CzcR in controlling efflux pump gene expression, which plays a critical role in the multi-drug resistance phenotype of *P. aeruginosa (* Chen et al., 2023 *)*. Additionally, Sheng et al. uncovered a novel resistance mechanism whereby insertion sequences (ISs) integrate into Cas genes, resulting in the inactivation of CRISPR-Cas systems. This insertional mutagenesis effectively transforms a bacterial defense mechanism, impairing its function. Their findings underscore the role of OS elements in disrupting CRISPR-Cas integrity, as demonstrated in *E. coli* isolates (Sen and Mukhopadhyay, 2024). Furthermore, Locus Biosciences (Morrisville, NC, USA) carried out the first clinical trial employing a CRISPR-Cas3 system delivered via phage to target *E. coli* causing urinary tract infections, yielding favorable safety and tolerability results (Kim et al., 2024).

**Figure 7:**
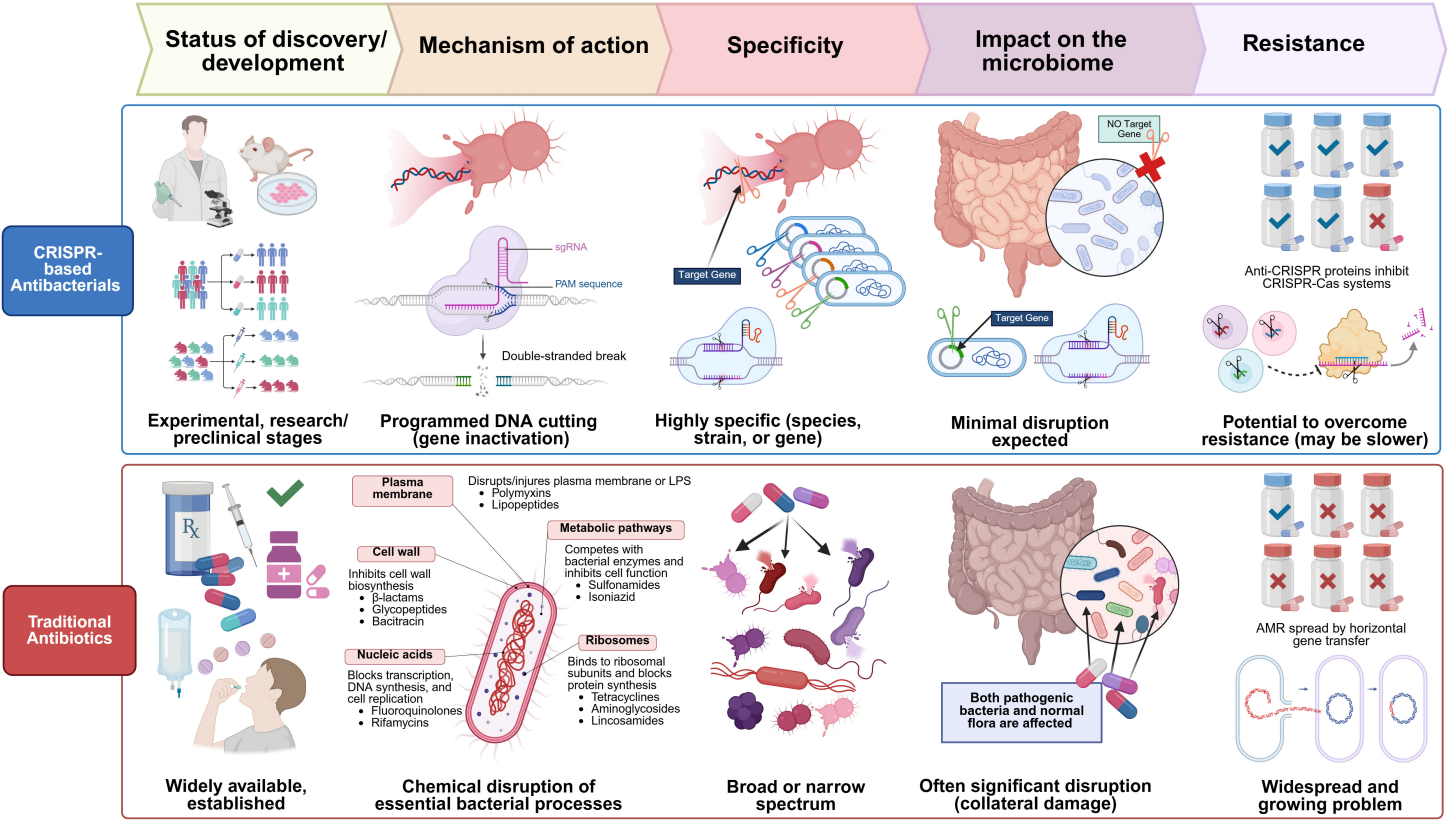
Comparative overview of CRISPR-based antibacterials and traditional antibiotics across five key aspects: development stage, mechanism of action, specificity, microbiome impact, and resistance. Traditional antibiotics, widely available and clinically established, exert their antibacterial effect through chemical disruption of essential bacterial processes, including inhibition of cell wall synthesis, protein translation, and DNA replication. These drugs display either broad or narrow-spectrum activity but often lead to significant collateral damage to the host microbiota. Furthermore, the widespread emergence of antimicrobial resistance (AMR), frequently driven by horizontal gene transfer, poses a major clinical challenge. In contrast, CRISPR-based antibacterials are in early research and preclinical stages. They function via sequence-specific, RNA-guided DNA cleavage, enabling precise gene inactivation. Their high specificity allows targeting of particular bacterial species or even strains, minimizing off-target effects and preserving the host microbiome. Although anti-CRISPR proteins may confer resistance, the potential to overcome such resistance exists, limiting resistance to CRISPR-based antibacterials.

Finally, it is important to consider the advantages of CRISPR-Cas-based antimicrobials over traditional antibiotics, as depicted in **Figure 7**. The development of CRISPR-Cas systems as versatile antimicrobials holds significant promise, allowing for the targeted attack of pathogens, even those resistant to conventional antibiotics. These intelligent tools can be tailored to disrupt essential bacterial genes, virulence factors, or specific antibiotic resistance genes, potentially reestablishing antibiotic efficacy or directly eradicating harmful bacteria while safeguarding beneficial microbiota (Uddin et al., 2021). Scientists have recently harnessed these powerful biological tools that hold enormous potential to treat infectious diseases, potentially enabling the development of more targeted antibiotics that solely attack disease-causing bacterial strains. This is linked to the unique mechanism of action and selectivity of these gene editing tools over broad-spectrum antibiotics, which can damage a wide range of bacteria, including the beneficial microbiota (Mayorga-Ramos et al., 2023).

### 5.4 Precision editing by next-generation CRISPR tools: base editors and prime editors

CRISPR-Cas systems can be adapted for a wide range of applications, including base editing and prime editing.

Base editing is evolving as a precise alternative to traditional CRISPR-Cas9. It signifies a groundbreaking breakthrough in genome editing technology, enabling accurate nucleotide substitutions. They comprise a nuclease-deficient Cas9 (usually nCas9 or dCas9) linked to a DNA deaminase enzyme. The Cas component directs the editor to the target location through an sgRNA, while the deaminase chemically alters a specific base within a small editing window close to the target site. This allows the deaminase to introduce specific point mutations into DNA without creating double-strand breaks (DSBs) or relying on donor DNA templates and repair efficiency (Wang et al., 2022). Early clinical translation efforts are focusing on diseases where precise edits of specific mutations are needed. Key therapeutic uses include rectifying harmful point mutations in genetic disorders, as shown in clinical trials for hypercholesterolemia by targeting PCSK 9 (Hoekstra and Van Eck, 2024), and HbF gene in sickle cell disease (Zeballos and Gaj, 2021). Recent advancements showcase dual-function editors capable of implementing specific combinations of nucleotide alterations simultaneously, along with engineered variants that exhibit markedly reduced off-target activity (Liang et al., 2022). These features establish base editing as a revolutionary technique in both research and therapeutic genomic medicine.

Prime editors signify a notable advancement in precision genome editing technology, which is more versatile than base editors. This technique allows for improved flexibility by integrating new genetic sequences into the genome with accuracy (Zeballos and Gaj, 2021). This search-and-replace system can execute all possible base-to-base conversions, small insertions, and precise deletions with fewer off-target effects compared to traditional CRISPR methods (Anzalone et al., 2019). It is capable of addressing multiple mutations without requiring donor templates and without generating direct DSBs. Prime editors comprise a Cas9 nickase attached to a reverse transcriptase (RT) enzyme. Guided by a specialized prime editing guide RNA (pegRNA), this system not only identifies the target site but also includes an RT template sequence that encodes the desired modification. The process starts with the nCas9 making a cut in one DNA strand at the target location, followed by pegRNA binding to the nicked strand. The RT then utilizes the pegRNA template to synthesize a new DNA strand incorporating the edit. Ultimately, cellular mechanisms resolve the intermediate structure to integrate the change into both DNA strands (Xu et al., 2024b).

Noteworthy therapeutic applications include rectifying pathogenic mutations in multiple diseases, such as cystic fibrosis (Bulcaen et al., 2024), Duchenne muscular dystrophy (Happi Mbakam et al., 2022), and metabolic disorders like phenylketonuria (Brooks et al., 2023). Recently, the FDA approved the use of prime editing in a phase 1/2 clinical trial for pediatric and adult patients with chronic granulomatosis disease, a rare immunodeficiency resulting from mutations in genes that impact phagocyte function (Biotechnology, 2024).

## 6 Hurdles in using CRISPR–Cas technology for therapeutic purposes

CRISPR has been recognized as a versatile and adaptable tool for molecular and clinical research and gene therapy approaches (Saber et al., 2020). It has been used in drug resistance research applications, such as gene function screening, resistant model creation, and molecular mechanism exploration (Yang et al., 2021). However, scientific, economic, and regulatory challenges face new genomic technologies, including CRISPR technology (Pacia et al., 2024). Several concerns confront this groundbreaking tool for genome editing:

### 6.1 Safety concerns

The main downside of CRISPR is its off-target cleavage of DNA sequences (Balon et al., 2022), which can decrease the efficiency of gene editing (Yang et al., 2021). The off-target effect occurs when Cas acts on untargeted genomic loci, leading to random cleavage at undesired sites, which can cause several adverse outcomes. These sites are often gRNA-dependent, since Cas9 can tolerate up to 3 mismatches between gRNA and genomic DNA (Guo et al., 2023). Modifying Cas9 and optimizing the gRNA can help mitigate off-target effects (Balon et al., 2022). Indeed, designing specific gRNAs for CRISPR-Cas systems can be performed with the aid of artificial intelligence (AI) models, which can increase the precision, specificity, and efficiency by predicting off-target scores (Dixit et al., 2023; Dai et al., 2024). Engineering Cas9 proteins can help to improve their specificity in binding to gRNA-matched genomic regions, ensuring perfect guide-target complementarity. Furthermore, these engineered variants of Cas9 have fewer mutagenic and immunogenic adverse effects (Kovalev et al., 2024). Another limitation of the CRISPR technique is the requirement for a PAM near the DNA target site. Efforts have been made to develop engineered nucleases that require less or no PAM. Recent research has shown that PAM-relaxed variants of Cas9 are improved by increased specificity and activity (Kovalev et al., 2024).

**Figure 8:**
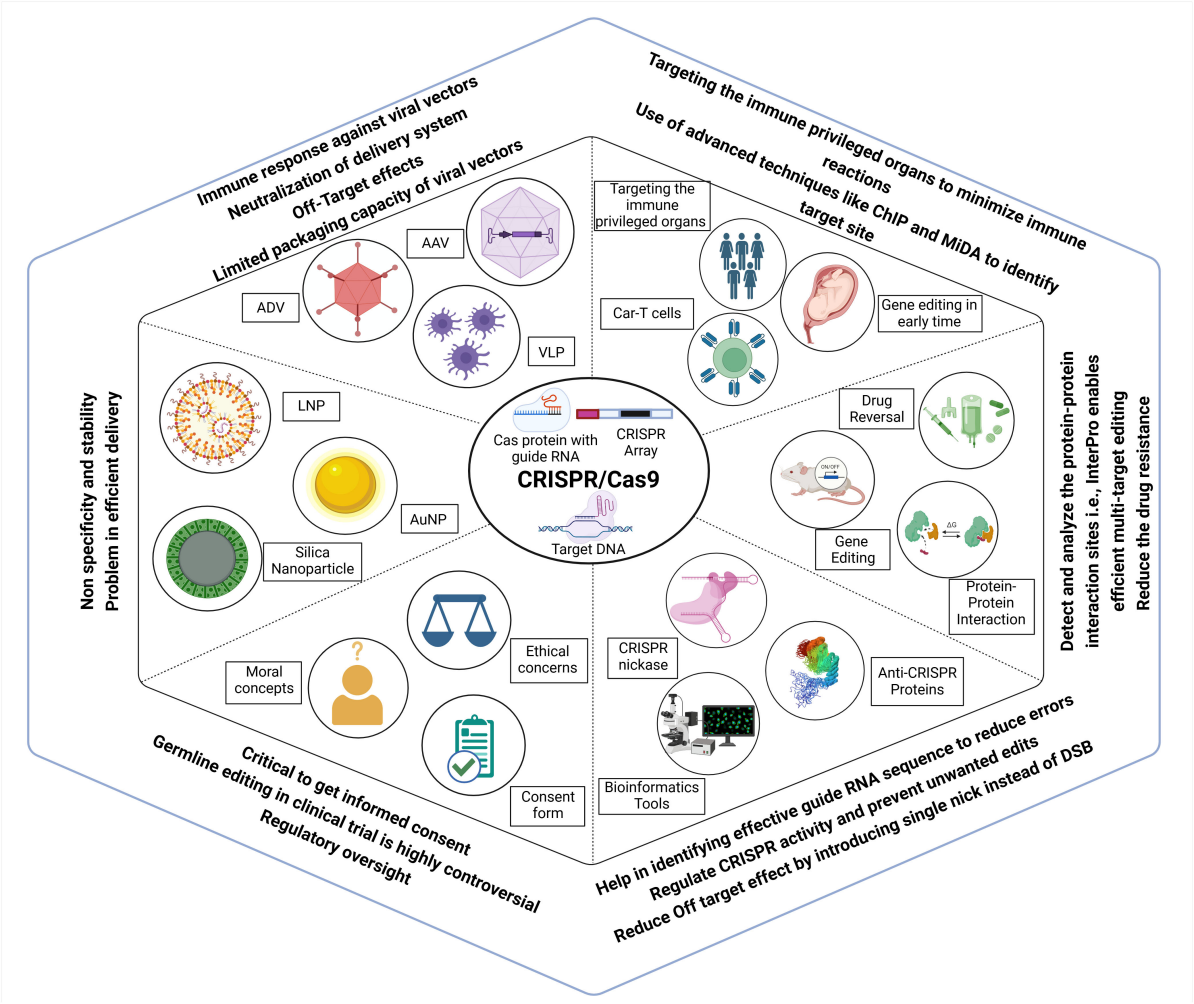
Challenges, ethical considerations and advancements associated with CRISPR Cas genome editing. Efficient delivery of CRISPR components remains a major hurdle, with vectors such as AAV, ADV, VLPs, and various nanoparticles limited by immune responses, off-target effects, and low stability. These issues can be mitigated by targeting immune-privileged organs, using early-stage gene editing, and applying advanced site identification tools. CRISPR application extends beyond gene correction to include drug resistance reversal, modulation of protein-protein interactions, and multi-target effects, and avoiding double-strand breaks. AI and bioinformatics tools assist in designing effective guide RNAs to enhance target specificity and minimize unwanted edits. However, germline editing remains highly controversial, requiring strict regulatory oversight, ethical reflection, comprehensive informed consent, and controlled clinical trials.

Gene therapy has the potential to cause immunotoxicity. The bacterial origin of the CRISPR system can trigger immunogenicity as it can be recognized as foreign by the human immune system (Ewaisha and Anderson, 2023). Selection of non-cross-reactive CRISPR types from non-ubiquitous organisms can be considered to overcome the effect of pre-existing immunity. The immune system may also react to the Cas9 protein, leading to the elimination of genetically modified cells. Further work is necessary to develop a remedy, such as using a recombinant Cas9 protein that blocks T-cell activation as described in **Figure 8**.

Viral vectors used for delivery can induce an adverse immune response, which poses a significant challenge. For example, adenoviruses, widely used viral vectors for the delivery of gene therapy (Wold and Toth, 2013), can induce cross-reactive adaptive immune responses with different serotypes of the same virus (Kovalev et al., 2024). Viral vector tissue tropism can be used for targeting desired disease sites, lowering the risk of systemic immune responses. *In silico* prediction of the immunogenicity of CRISPR therapeutics can be helpful in light of the advances in various tools that can leverage AI for efficient design with fewer adverse reactions. Enabling the effective and secure transportation of the CRISPR–Cas9 system into targeted cells presents a significant challenge (Balon et al., 2022).

One additional challenge that this innovative technology faces is the lack of secure and effective distribution channels; thus, safe delivery methods are required. CRISPR delivery employs three main categories of carriers: physical transfer, viral vectors, and non-viral vectors (shown in **Figure 8**). Microinjection and electroporation are well-established techniques used for the physical delivery of drugs, and ongoing scientific research is focused on hydrodynamic delivery systems (Zhang et al., 2021c). Adeno-associated viruses (AAVs), full-sized adenoviruses, and lentiviruses are commonly used as viral vectors to transport genetic material. Viruses serve as the primary vehicle for delivering CRISPR–Cas9 into living organisms. Non-viral vectors do not receive the same level of awareness as viral vectors, but they provide unique advantages that have attracted scientific research (Shirani-Bidabadi et al., 2023). Scientists are creating nano-carriers that specifically transport CRISPR–Cas9 to cancerous tumors. Self-assembled nanoparticles are chosen for their ability to pack and protect efficiently. Nano-carriers possess the ability to contain reactive molecules, hence enhancing the processes of circulation, absorption, and targeting. Additionally, they can exhibit delivery patterns that are sensitive to stimuli, so allowing gene editing. Nanotechnology has improved the effectiveness of cancer therapy and minimized the unwanted consequences of CRISPR–Cas9 treatment (Shirani-Bidabadi et al., 2023).

### 6.2 Ethical concerns

There are ethical concerns when implementing CRISPR–Cas technology in preclinical or clinical trials. The primary issue is related to apprehensions over off-target effects, adverse consequences, inadequate editing, and lower efficacy of the treatment in comparison to conventional therapeutic drugs (Kotagama et al., 2019). Furthermore, one of the most contentious ethical issues is the potential for using CRISPR technology in human germline editing (shown in **Figure 8**), which entails making alterations to sperm, eggs, or embryos that can be inheritable. This situation raises concerns regarding unforeseen and undesirable consequences, as well as the moral implications associated with the modification of human genetics (Ben Ouagrham-Gormley and Fye-Marnien, 2019).

In 2017, Ma et al. reported the first therapeutic germline intervention, which involved creating modified zygotes by fertilizing healthy oocytes with sperm from a carrier of a mutation linked to hypertrophic cardiomyopathy. Utilizing CRISPR–Cas9, they corrected the genetic defect in these zygotes, resulting in mutation-free, viable embryos (Ma et al., 2017). This germline intervention was conducted for research purposes, demonstrating the feasibility of correcting gene mutations in viable human embryos through genome editing techniques, and also proved the efficiency, accuracy, and safety of this method (Rubeis and Steger, 2018). This method can also be used for other inheritable traits as a way to prevent diseases. As a potential life-saving treatment, it offers advantages that surpass its risks. So far, no clinical trials involving humans have taken place, and the embryos produced were solely for research, not for implantation in the womb. The ongoing debate centers on the moral implications of modifying genomic material in a way that these edits could be passed on to future generations (Brokowski, 2018).

Notably, a Chinese research team conducted an unauthorized trial involving germline editing using CRISPR-Cas9 to create embryos resistant to HIV from an infected father. In 2018, Dr. He Jiankui sought to alter both copies of the CCR5 gene, allegedly intending to render the future babies’ white blood cells incapable of contracting HIV. Consequently, two twin girls were born with genetically modified traits that grant them immunity to HIV. There are also concerns that modifications to the CCR5 gene may impact brain development and potentially enhance cognitive abilities (Raposo, 2019). This was not part of an approved clinical trial, resulting in severe backlash and his incarceration. In December 2019, the Chinese doctor was convicted of illegal medical practices and received a three-year prison sentence (Alonso and Savulescu, 2021).

Before starting any clinical trial focused on human germline editing, it is vital to gather extensive preclinical safety data, conduct ethical assessments, obtain regulatory approvals, and achieve wide societal consensus. Establishing global ethical standards and guidelines is imperative for managing CRISPR applications, particularly in the context of human germline editing. This requires working together with international organizations to maintain biosecurity and prevent misuse (Dieuliis and Giordano, 2018). Continuous research and open dialogue among scientists, ethicists, and policymakers are essential for addressing the ethical and safety challenges associated with CRISPR technology. This collaborative approach guarantees that the technology is utilized in a responsible and safe manner (Aljabali et al., 2024).

### 6.3 Resistance progression against CRISPR-Cas

CRISPR technology faces resistance challenges both in human applications and bacterial systems. The emergence of resistance poses a significant challenge to the effective application of CRISPR-Cas technologies. Bacteria use various mechanisms to defend against CRISPR, which play a key role in the continuous co-evolutionary battle between prokaryotes and their viral predators, impacting the advancement of CRISPR-based antimicrobials significantly (Uribe et al., 2021).

In the context of phage interactions, resistance can rapidly arise through the accumulation of point mutations within the target sequence of the CRISPR-Cas system (Tao et al., 2018). A similar phenomenon may occur when targeting antibiotic resistance genes, particularly under conditions of positive selection, such as the presence of antibiotics for which the resistance gene provides protection. Furthermore, resistance may develop through the disruption or deletion of essential Cas genes, impairing the ability of the system to cleave target DNA, or through the loss of targeting spacers critical for CRISPR functionality (Common et al., 2020). Apart from point mutation, resistance can also arise through the selection of anti-CRISPR genes (Kadkhoda et al., 2024). These genes encode small proteins capable of binding and inhibiting key components of the CRISPR-Cas immune machinery. Till now, more than 50 unique families of anti-CRISPR (*acr*) genes have been identified, targeting both type I and type II CRISPR-Cas system (Chaudhary et al., 2018).

Another cause of resistance against CRISPR-Cas system is the microbial ecosystems present in the environmental niches, as well as within human hosts (Watson et al., 2023). These microbial ecosystems are extraordinarily diverse and structurally intricate. These natural microbial communities form unique, distinct microbiomes, each comprising billions of bacterial cells representing thousands of species per gram of sample. Such diversity poses a considerable challenge when deploying CRISPR-Cas system for combating antimicrobial resistance. Although CRISPR-Cas technologies have shown significant promise in eradicating pathogenic bacteria or restoring antibiotic sensitivity in resistant strains, current research has primarily been confined to simplified, near-clonal bacterial populations. Only a limited number of *in vivo* investigations, mostly utilizing murine models, have attempted to target Gram-negative pathogens within the gastrointestinal tract to impede their colonization (Geinoro et al., 2024).

Moreover, anticipating the ecological consequences of CRISPR-Cas antimicrobial interventions within complex microbial communities remains a substantial hurdle. Targeted depletion of a specific strain may inadvertently disrupt microbial homeostasis (dysbiosis), creating ecological niches that can be exploited by opportunistic or harmful species. As a result, the broader implications of eliminating antimicrobial resistance within these multifaceted microbial ecosystems must be rigorously assessed prior to the clinical or environmental application of CRISPR-Cas based antimicrobials (Kadkhoda et al., 2024).

Similarly, human cells can show resistance to CRISPR, often noted as low editing efficiency that leads to unintended outcomes due to the poor interplay between the editing tool and the human cell. As a result, only a small fraction of the target cell population displays the desired genetic modification. This can be attributed to DNA repair pathway alterations, as cells can modify their DNA repair mechanisms in ways that reduce CRISPR editing efficiency (Liao et al., 2024). Additionally, the activation of p53-mediated responses may be another contributing factor. CRISPR-induced DNA double-strand breaks can activate p53, a tumor suppressor protein, leading to cell cycle arrest or apoptosis. This process effectively favors the survival of cells with impaired p53 pathways, enabling them to divide even in the presence of DNA damage (Conti and Di Micco, 2018). Pre-existing genetic variations also play a role, as the natural genetic polymorphisms in target sequences or PAM sites can prevent CRISPR recognition and cutting (Hirakawa et al., 2020). Editing may not take place in all cells within a population or even on all alleles in a single cell, resulting in a mosaic mixture of edited, partially edited, and unedited cells. This incomplete editing can compromise therapeutic efficacy, particularly if a high percentage of corrected cells is essential for phenotypic rescue (Hirakawa et al., 2020; Yang et al., 2021).

## 7 Strategies for overcoming the obstacles to enhance the delivery of CRISPR–Cas

Multiple challenges have emerged in implementing the CRISPR–Cas system in medical microbiology applications. Therefore, several methodologies have been examined and evaluated to tackle these issues.

An approach to enhance the delivery of CRISPR–Cas involves the use of conjugative plasmids, which can be further enhanced by screening for additional plasmids with a broader range of hosts. The researchers intentionally chose conjugative plasmids from the *Enterobacteriaceae* family to target pathogenic bacteria in the gut. The researchers discovered plasmids that exhibit improved DNA transfer efficiency within the gut microbiota. Consequently, scientists have developed a genetically engineered probiotic strain capable of utilizing exceptionally effective plasmids for transmitting the CRISPR–Cas system. This strain can eradicate chloramphenicol-resistant *E. coli* germs in mice (Neil et al., 2021). Conjugation is a less effective method of removing resistant species or those with specific genetic traits than phage delivery, nevertheless, it can still be more effective in some cases. This is because certain conjugative plasmids have the capacity for replication and a wide range of hosts. It is essential to design a delivery system that works with a broad variety of hosts, especially when applying the CRISPR–Cas system in complex microbial communities. Because the reaction of bacterial groups to this application is unknown, careful monitoring and analysis of the ecological consequences of the CRISPR–Cas system are essential. With the help of this study, researchers will be able to better comprehend how the removal of drug-resistance genes affects the frequency of other bacterial species in the community. Phage engineering shows promise as a strategy to overcome the constraints of current phages in delivering CRISPR–Cas antimicrobial agents. Scientists have integrated the CRISPR–Cas system into the genetic material of a phage, resulting in a modified bacteriophage. This system has two benefits: first, is the phage’s specific affinity for the target initiates the demise of pathogenic cells by employing an abortive infection pathway during phage invasion. Secondly, the introduction of the CRISPR–Cas system into the specific pathogen results in the removal of the target gene and induces apoptosis in the harmful bacteria (Shukla et al., 2021). Researchers have used this technique to eradicate multidrug resistance genes from *K. pneumoniae* by modifying the *Klebsiella* virulent bacteriophage phiKpS2. However, administering the CRISPR–Cas system using phages does not precisely mimic phage therapy and has similar challenges. Furthermore, the application of a phage system that incorporates CRISPR–Cas to restore susceptibility in antibiotic-resistant bacteria by antibiotic therapy results in the eradication of antibiotic-resistant mutants that evade treatment. Yosef et al. have developed a method that uses temperate phages to deliver the CRISPR–Cas system into bacterial cells to address the challenges posed by antibiotic-resistant bacteria. The CRISPR spacers utilized in this method are precisely designed to selectively target genes that are associated with antibiotic resistance and lytic phages. When the CRISPR–Cas phage system is introduced into the samples, bacteria that are capable of integrating phage DNA into their own DNA restore their susceptibility to antibiotics. On the other hand, bacteria without this capability are susceptible to the damaging actions of lytic phages (Kundar and Gokarn, 2022). Integrating several phages to stop the creation of resistant mutants and improve the effectiveness of treatment approaches is feasible since pathogenic bacteria can evolve and become resistant to manufactured phages.

However, the anti-CRISPR activity of *acr* genes can be countered by some CRISPR–Cas systemic changes that can negate the effects of anti-CRISPR proteins. It is also feasible to produce CRISPR variants that are insensitive to Acr proteins to solve the problem of accessing Acr proteins. Similarly, several strategies can improve the toxicity and efficacy of Cas9 nucleases in various bacterial hosts, such as mixing alternative Cas proteins with Cas9.

Furthermore, a large repertoire of strategies has been developed to improve specificity (high-fidelity Cas, better gRNAs), favoring precise repair (base/prime editing), developing stealthier delivery systems, and managing host immune responses. Combating bacterial resistance, particularly for CRISPR antimicrobials, requires anticipating evolution through multiplex targeting, careful target selection, and robust delivery methods capable of overcoming bacterial defenses while addressing the threat of Acr proteins. Vigilance regarding off-target effects, immunogenicity, and the emergence of resistance remains paramount as these technologies move further into clinical settings. Utilizing current technological advancements could aid in overcoming challenges related to obtaining regulatory approval for the CRISPR–Cas system’s implementation in the real world (Kundar and Gokarn, 2022).

## 8 Conclusions and future perspectives

Undoubtedly, CRISPR has become a valuable gene-editing tool offering unprecedented precision and versatility in manipulating genetic material, although there is still much to learn about it. CRISPR–Cas gene editing technology has a wide range of valuable applications, which could potentially replace conventional treatments and has the potential to advance next-generation diagnostic platforms. Its future perspectives are vast and promising, spanning various fields in medicine. There is enough interest in the field to support the establishment of several biotech start-ups aiming to treat human diseases with CRISPR-inspired technologies. The future use of CRISPR–Cas technologies offer significant potential for both medical applications and genome editing in the majority, if not all, species. As we progress from modifying the genomes of model organisms to humans, numerous ethical issues must be considered; thus, it is crucial to carefully consider both the benefits and challenges associated with this groundbreaking tool. We are only beginning to grasp how the CRISPR revolution will impact our future. The future development of this technology will allow for a more personalized approach to targeted treatment for various illnesses, but indeed, this needs more research. Therefore, conducting more thorough preclinical and clinical trials before implementing this technology in human treatments is essential.
